# Supplementary material for: Changing diagnostic criteria for gestational diabetes (CDC4G) in Sweden: A stepped wedge cluster randomised trial
Source: PLoS Med. 2024 Jul 8;21(7):e1004420. doi: 10.1371/journal.pmed.1004420 (PMC11262657; doi:10.1371/journal.pmed.1004420)
Supplement: S1 Appendix — (PDF) [file pmed.1004420.s002.pdf]

Research plan- Helena Fadl

The CDC4G trial:
Impact of Changing Diagnostic Criteria for Gestational diabetes in Sweden- a stepped wedge randomized trial

Purpose and aims

The overall aim of this national, randomized trial is to evaluate the impact of the new diagnostic criteria for gestational diabetes (GDM) on maternal and neonatal outcomes in Sweden.

Since June 2015, the National Board of Health in Sweden adopted the WHO criteria for the diagnosis and treatment of GDM. The new criteria are based on a 75% excess risk of adverse child outcome and include lower cut-off levels for GDM diagnosis.There is clear evidence that treatment of GDM is beneficial, however, data on the impact of different diagnostic criteria for GDM on risks of adverse pregnancy outcomes are scarce. The few randomized trials that exist have been performed in different settings and using varying diagnostic criteria, and data on a population level do not exist. Thus, there is an urgent need for a population-based evaluation of the individual, clinical and economic consequences of the new diagnostic criteria for GDM.

The purpose of the project is therefore to evaluate the impact of the new guidelines by the National Board of Health regarding diagnosis of GDM, in Sweden. Today about 1-3% of pregnant women are diagnosed with GDM. With the new guidelines the prevalence of GDM is expected to increase to 15-20% as shown in other countries. Such an increase in diagnosed cases will have great implications on clinical, individual and economic aspects as those women need extensive attention from health care professionals. The initiative for this implementation study came from the expert panel in The National Board of Health working group for the new guidelines.

The underlying hypothesis I: is that treating women with GDM defined by the new criteria will reduce risks of adverse pregnancy outcomes in the Swedish population.

Hypothesis II: the new criteria will be cost effective. Even though there will be more costs in obstetrics, reduced costs will be seen in neonatal care and probably later on in primary health care.

The aim of the CDC4G trial is to test whether there is a reduction in adverse maternal and neonatal outcomes following introduction of the new GDM diagnosis criteria and to assess the impact on health economy.

Our specific objectives are:

1. To compare the rates of adverse maternal and neonatal outcome before and after implementation of the new guidelines for diagnosis of GDM.
2. To compare the health costs before and after the changed criteria and assess the net cost/saving.
3. To assess the adverse outcomes and health costs using the new WHO criteria (75% excess risk) and the criteria based upon the 100% excess risk of neonatal adverse outcomes (5.3, 10.6, 9.0 mmol/l respectively).
4. To create a prospective cohort to compare the long term health effects for mother and children using the old and new diagnostic criteria.

Research plan- Helena Fadl

Background:

GDM is a growing problem globally with increased risks for both mother and baby during and after pregnancy (1-5). Treatment of hyperglycemia reduced the rate of LGA and preeclampsia, and lifestyle programmes has been shown to be effective in preventing type 2 diabetes. Every year 100 000 women receive maternity care in Sweden, of which 1% have been diagnosed with GDM using the old guidelines, se below (4,5). The need of a national GDM screening and diagnosis programme has recently been underpinned by the 2013 WHO recommendations. These include some form of screening (either risk factor or universal testing), followed by a 2 hour, 3 time point blood testing (fasting, one hour, 2 hour), 75g oral glucose tolerance test (OGTT) (6). In Sweden there is no uniform screening strategy, so there is a need to harmonize diagnostic criteria so that uniform screening strategies can be implemented, according to National guidelines in Sweden.

New criteria have been introduced that acknowledge the importance of the fasting and 1 hour blood testing for predicting adverse pregnancy outcomes. The current Swedish GDM criteria include a fasting glucose test of ≥7.0 mmol/l and/or 2 hour glucose threshold of ≥8.9 mmol/l venous or ≥10.0 mmol/l capillary blood. The 2013 WHO criteria define GDM as ≥5.1, ≥10.0 and/or ≥9.0 mmol/l fasting, 1 hour and/or 2 hour cut offs respectively based upon a 75% excess risk of adverse neonatal outcomes (eg large for gestational age LGA infants).

The WHO criteria are based on data from the HAPO study using venous plasma from over 25,000 women (7). The merits of the new criteria are still questioned as the prevalence of GDM will increase significantly. However, there is consensus regarding the use of venous plasma measured at three time points (fasting, 1 and 2h post-load). Additionally, the cost and clinical effectiveness of the new criteria are under debate (8-11). To meet the concern of costs versus clinical effectiveness, another option could be to use criteria based upon a 100% excess risk of adverse neonatal outcomes instead of the 75 % excess risk these new guidelines are based on.

In June 2015, the Swedish National Board of Health reviewed the evidence on the current Swedish and WHO GDM criteria and recommended adoption of the new, stricter WHO cut offs (12). One argument for changing the diagnostic criteria is to allow international comparisons.The board recommended that every county council should include these criteria in their clinical guidelines and decide how to implement the relevant changes locally. In this project our ambition is to enable all Swedish maternity clinics to implement a national standardized and harmonized practise in screening and diagnosis of GDM.

With the current variation in GDM screening/diagnostic practice across Sweden (13), and the debate over the criteria, there was a recognition that the shift to the recommended new guideline could be either by an ad hoc or planned and structured way. In addition, the national registries in Sweden offer a unique possibility to assess potential impacts of the new criteria on pregnancy outcomes and long term health for both mother and child. There was therefore a widespread agreement that the switch should be managed using a randomised roll out format: either as a cluster randomised controlled trial, meaning that at the end of the trial, some sites would not be compliant with the Swedish National Board of Health recommendation, or as a stepped wedge randomised controlled trial (14-18). The latter would result in all participating sites adopting the new criteria by the end of the trial.

To our knowledge, the “stepped wedge” trial design has not yet been used in Sweden, and this study design could be a new way of evaluating clinical management scientifically. We consider this study design superior to cluster randomization since management of GDM may vary over time and between clinics.

Research plan- Helena Fadl

Project description

Hypothesis

Treating women with GDM defined by the new criteria will reduce risks of adverse pregnancy outcomes in the Swedish population.

The health care costs will be reduced overall.

Method

Study design

A national prospective stepped wedge randomised controlled trial. The stepped wedge cluster randomised design starts with a period of baseline collection, with all clusters (clinics) still using their old GDM criteria. Subsequently, at periodic time points called "steps", a cluster crosses to the new GDM criteria in a randomised order while the remaining clusters continue to use the old GDM criteria until all clusters have crossed to the new GDM criteria. To clarify the design it is described in PICO terms below:

P: The population is all pregnant women attending maternal health care in risk for GDM

I: The intervention is switching to new diagnostic criteria for GDM

C: The comparison is done before and after intervention, that is, comparing old and new criteria

O: LGA is the main outcome since the treatment of GDM gives a significant reduction in rates of LGA. Other outcomes are also shown to be associated with different levels of hyperglycemia, but randomized trials have been too small to show the treatment effect in rare outcomes.

Population and sample size

All pregnant women treated in the participating hospitals will be included in the study. Today 15 of the 40 Swedish maternity clinics intent to participate in the study (Örebro, Södersjukhuset, Danderyd, Uppsala Akademiska sjukhuset, Sahlgrenska Gothenburg, Malmö, Lund, Helsingborg, Halmstad, Kristanstad, Norra Älvsborgs sjukhus, Västerås, Eskilstuna, Karlstad and Nyköping). Most likely, even the rest of the Stockholm clinics will join. These clinics cover more than 40% of the yearly Swedish pregnant population. All clinics in Sweden are invited and we expect at least 20 hospitals to contribute to patient inclusion when the study is launched. The minimum sample size is n= 32490 (please see the Statistical calculations) which is expected to be reached within 8 -10 months after study start.

Recruitment, selection of participants and randomization

The intervention in this study is the switch to the new GDM diagnosis criteria as part of the routine care of any participating health service. No extra patient data will be collected than what is already registered in the national quality and health registries. Therefore all women attending participating antenatal health services (primary care and hospital) will be part of the study population. However, the national Pregnancy register is a quality register and thus voluntary so that women always have the option to opt out. Also, women are always entitled to decline testing and, if GDM is diagnosed, decline treatment.

Randomization will be done by Statistician University Clinic Örebro; and each centre will be informed about timing of switching regime 3 months before starting time.

Inclusion criteria

Research plan- Helena Fadl

All women under the care of a participating health service (primary care and hospital) will be included.

Informed consent
Informed consent will not be requested beyond the invitation to opt out of the Pregnancy Register (a routine today) and the option to refuse any aspect of management at any time. We believe this is justifiable on the basis of:

a) This is a Swedish National Board of Health policy directive and hence management of GDM is changing as a result of the new national recommendation.
b) No additional questionnaires or samples are being requested
c) Women always have the right according to Swedish Law to change clinic and refuse any aspect of care

Data sources
National Pregnancy, Diabetes and Child Health Registers.

Pregnancy Register data: All variables within the Pregnancy register will be accessible for the analyses.

Diabetes Register data: All variables within the Diabetes register will be accessible for the analyses. Data linkage will be performed by the National Board of Health using the Personal Identification Number approximately every 3 years after the first 12 months.

SNQ, Swedish neonatal Quality register: Variables on children under neonatal care for the study population.

Swedish health care registers: All variables within the Swedish health care registers will be accessible for the analyses (e.g. the Swedish inpatient register, death register, cancer register, medication register).

- Clinic data: Screening methods used (universal, risk factor (which), random glucose and criteria), Motivational interviewing skills (all, some, none) will be collected through the Pregnancy registers structural yearly reporting and through collecting information through PI:s. Blood glucose data on OGTT will be collected in the Pregnancy register and blood glucose values for the mother and child through patient records if not transferred to the Pregnancy register.

Primary and secondary outcomes

Primary outcome
- Large for Gestational Age (LGA), defined as a birth weight > the 90th percentile for gestational age and sex

Secondary outcomes
- Composite of severe adverse outcomes (stillbirth, neonatal death, Erbs palsy, metabolic acidosis defined as pH <7.05 and BE >12 mmol/l in umbilical artery or pH <7.0 in umbilical artery, Apgar score <4 at 5 minutes, HIE I-III, intracranial haemorrhage, neonatal convulsions, meconium aspiration syndrome, mechanical ventilation)
- 5-Min Apgar score <7

- Fractured clavicle
- Blood glucose in the infants
- Prematurity <37 weeks
- NICU admission yes/no
- NICU days
- Small for Gestational Age
- Health economic outcome
- Incremental Cost Effectiveness Ratio
- Hypoglycaemia needing IV therapy
- Phototherapy
- Blood glucose in the infants

Secondary maternal outcomes

- Hypertension, Pre-eclampsia-defined using Swedish ICD coding (O139, O14, O15)
- Shoulder dystocia
- Induction of labour
- Emergency CS
- Elective CS
- Intrumental delivery
- Length of maternal postnatal stay
- Perineal trauma-3 and 4 degree
- Breastfeeding at hospital discharge
- Shoulder dysctocia
-

Statistical power calculation

Preliminary sample size estimation was performed for a stepped wedge cluster randomised design using STATA release 14. With 18 clinics (clusters) participating and an intra cluster correlation (ICC) of 0.0018 a total sample size of 32480 pregnant women (16245 before change and 16245 after change of the new GDM criteria) have 80% statistical power to detect a risk reduction of LGA by 1.5% on a population level. The ICC was estimated from the variation of LGA incidence between participating clinics from previous year.

Statistical methods

To statistically evaluate LGA incidence difference between old and new GDM criteria a logistic regression model with random effects for cluster and fixed effect for each step will be used. The statistical analysis will be performed by Scott Montgomery, Professor in epidemiology and Anders Magnusson, Statistician, Clinical Epidemiology Research Center, region Örebro County.

Workplan

This is a 5-year project which is ready to be launched from April 1st 2017. By this time, all sites will have introduced the agreed GDM and obstetric management guidelines, switched to the use of venous blood for screening of GDM and developed a local staffing plan. Follow up of women by the diabetes and other national health registries is planned for 25 years after inclusion.

**Milestones**	
Start	April 1st 2017

50% randomised	August 1st 2017
Final wedge commences	March1st 2018
Primary analyses completed	September 31st 2018
First follow up analyses	September/fall 2019
Maternal diabetes/offspring health analyses	March 2023

Project organization
The principal investigator and participating researchers have a solid scientific background and experience of collaboration including the work in the expert panel of the National Board of Health guidelines on GDM. The study group represents competence in the fields of obstetrics, paediatrics, neonatology, clinical medicine, nursing, epidemiology, biostatistics, and health economy.

Principal investigator (PI)

The study will be coordinated by PI **Helena Fadl**, MD, PhD, Department of Obstetrics and Gynecology, Örebro University Hospital.

Co-Investigators

Participating researchers represent, so far, 17 of Swedens' Obstetrical departments in 6 County Councils (landsting) and Professor in medicine **David Simmons,** Western University, Sydney, guest Professor Örebro University.

Following participating researchers are in the steering group of the CDC4G-study:

Erik Schwarcz, MD, Assoc prof, Dept of Endocrinology, Region Örebro County

Stefan Jansson, MD, General practicioner, Brickebackens Health Care center, Region Örebro County

Lars Hagberg, Health economist, Assoc prof, University Hospital Research center, Region Örebro County

Kerstin Berntorp, Professor in Endocrinology, Malmö University Hospital

Ulla-Britt Wennerholm, MD**,** Assoc professor, Dept of Obstetrics, Sahlgrenska University Hospital Västra Götaland Region

Verena Sengpiel, MD**,** Assoc prof, Dept of Obstetrics, Sahlgrenska University Hospital Västra Götaland Region

Claes Ignell PhD, Dept of Obstetrics, Helsingborg, Region Skåne

Anna-Karin Wikström, MD, Prof, Dept of Obstetrics, Danderyd Hospital

Fredrik Ahlsson, Assoc prof, pediatrics, Uppsala University

Helena Strevens, Assoc prof, Dept of Obstetrics, Lund University Hospital

Karin Hildén, MD, PhD student, Dept of Obstetrics, Region Örebro County.

Ändrad fältkod

Scott Montgomery, Professor, Clinical Epidemiology Unit, University Hospital Örebro

Anders Magnuson, Statistician, Clinical Epidemiology and Statistical Unit, University Hospital Örebro

Carina Ursing, MD, PhD, Södersjukhuset, Stockholm

Elisabeth Storck-Lindholm, MD, PhD, Södersjukhuset, Stockholm

Research plan- Helena Fadl

Martina Persson, MD, PhD, Karolinska Institute, Stockholm

Annika Esscher, MD, PhD, Uppsala University

Maryam Saeedi, MD, research AT Region Örebro County march 2017 -2018.

Preliminary results

There are no preliminary results from Sweden, but from other European countries the rate of GDM increases up to 15-20%, with some health cost benefits, mainly do to reduced neonatal care costs . The change in LGA incidence on a population level has not been shown; other countries do not have full covered health registers to be able to measure these outcomes.

Dissemination

Publications will include the steering group and the local PI:s from each participating site. A health economics paper will be published. Publications will include:

- Statistical considerations of a stepped wedge randomised controlled trial with unequal clusters
- Trial protocol
- Baseline data
- Main trial findings
- Health economics paper including use of higher thresholds
- Follow up of maternal diabetes, offspring anthropometry at birth/major health issues and development at 12 months
- Follow up of maternal diabetes, offspring anthropometry at birth/major health issues and development at 4 years

Communication with public, patient organizations, policy makers etc. Each of the co researchers have National collaborations with different policy makers and the National Board of Health welcomes the results of this study for further evaluation and decision making.

International and national collaboration

The steering group consists of researchers from different parts of Sweden representing different geographical regions as well as specialties. We have a group with members from both Primary health care and Hospitals and both doctors and midwifes. A health economist is also part of the project group. As an international well known researcher, Prof David Simmons adds with his expertise in the area. He has been working with the WHO project group and has great experience about the clinical issues and problems in this area.

Prof David Simmons and PI Helena Fadl are both members of the DPSG; Diabetes and Pregnancy Study Group (www.dpsghome.org). This is a research group under EASD (European asscociation for the Study of Diabetes) that has a common interest in the research field of diabetes in pregnancy. The study will be discussed and introduced at the meetings of DPSG and can lead to European collaboration projects in the future.

Research plan- Helena Fadl

Grants

Local grants have been received from Region Örebro County, Nyckelfonden (268 000:-). Grants from VR has been applies, decision fall 2016. Grants have been applied from Diabetesfonden fall 2016, decision fall 2016. Several applications to local research funds are currently being filed.

Clinical significance

The study is important, not only for Sweden, but also for the international community. Sweden with its public health care system, close to 100% of all pregnant women participating in antenatal care and several national mandatory health care registers that can be linked by the personal identification number is the only country that can approach this clinical controversy by doing a randomized trial on the effects of changing clinical routines. The National Board of health supports this study.

This study could also be a good example on how to scientifically evaluate new treatment options in clinical work by the stepped wedge design. Randomized trials are usually smaller and effects on a population level are difficult (nearly impossible) to show. In this planned design and using the clinical quality registers that exist in Sweden we attempt to scientifically evaluate the much-debated change in diagnostic criteria for GDM in Sweden.

For Sweden, it is utterly important to evaluate these new criteria, so that the professionals can agree on equal care for these patients. Over the years, as long as screening and treating gestational diabetes has been done; there has been, controversies about the clinical management of the disease. The National board of health is supportive of this study, since the evidence is sparse on health economics and on population effects of the new recommended criteria for GDM.

For the individual, the results of this study will give better and more equal care for the >100 000 women attending maternal health care during pregnancy. The overall aim to reduce complications for the mother and child is an expected result. We consider this study to be of high priority; if this study is not performed the clinical controversy will continue in Sweden and elsewhere and there will be no evidence if the adverse pregnancy outcomes and the health costs associated with the new criteria can be defended.

Independent line of research

This study will not interfere with other research projects of the different collaborators.

References

1. Simmons D: Prevention of gestational diabetes mellitus: Where are we now? *Diabetes Obes Metab* 17:824-834, 2015
2. Fadl HE, Gardefors S, Hjertberg R, Nord E, Persson B, Schwarcz E, Aman J, Ostlund IK, Hanson US: Randomized controlled study in pregnancy on treatment of marked hyperglycemia that is short of overt diabetes. *Acta Obstet Gynecol Scand* 2015

3. Fadl H, Magnuson A, Ostlund I, Montgomery S, Hanson U, Schwarcz E: Gestational diabetes mellitus and later cardiovascular disease: A swedish population based case-control study. *BJOG* 121:1530-1536, 2014
4. Fadl HE, Ostlund IK, Magnuson AF, Hanson US: Maternal and neonatal outcomes and time trends of gestational diabetes mellitus in sweden from 1991 to 2003. *Diabet Med* 27:436-441, 2010
5. Ignell C, Claesson R, Anderberg E, Berntorp K: Trends in the prevalence of gestational diabetes mellitus in southern sweden, 2003-2012. *Acta Obstet Gynecol Scand* 93:420-424, 2014
6. WHO. Diagnostic criteria and classification of hyperglycaemia first detected in pregnancy. In: WHO Publications; 2013.
7. Metzger BE, Lowe LP, Dyer AR, Trimble ER, Chaovarindr U, Coustan DR, Hadden DR, McCance DR, Hod M, McIntyre HD, Oats JJ, Persson B, Rogers MS, Sacks DA: Hyperglycemia and adverse pregnancy outcomes. *N Engl J Med* 358:1991-2002, 2008
8. Chen PY, Finkelstein EA, Ng MJ, Yap F, Yeo GS, Rajadurai VS, Chong YS, Gluckman PD, Saw SM, Kwek KY, Tan KH: Incremental cost-effectiveness analysis of gestational diabetes mellitus screening strategies in singapore. *Asia Pac J Public Health* 28:15-25, 2016
9. Weile LK, Kahn JG, Marseille E, Jensen DM, Damm P, Lohse N: Global cost-effectiveness of gdm screening and management: Current knowledge and future needs. *Best Pract Res Clin Obstet Gynaecol* 29:206-224, 2015
10. Mission JF, Ohno MS, Cheng YW, Caughey AB: Gestational diabetes screening with the new iadpsg guidelines: A cost-effectiveness analysis. *Am J Obstet Gynecol* 207:326 e321-329, 2012
11. Gillespie P, O'Neill C, Avalos G, Dunne FP, Collaborators AD: New estimates of the costs of universal screening for gestational diabetes mellitus in ireland. *Ir Med J* 105:15-18, 2012
12. Socialstyrelsen: Gränsvärden för graviditetsdiabetes. 2015
13. Lindqvist M, Persson M, Lindkvist M, Mogren I: No consensus on gestational diabetes mellitus screening regimes in sweden: Pregnancy outcomes in relation to different screening regimes 2011 to 2012, a cross-sectional study. *BMC Pregnancy Childbirth* 14:185, 2014
14. Brown CA, Lilford RJ: The stepped wedge trial design: A systematic review. *BMC Med Res Methodol* 6:54, 2006
15. de Hoop E, van der Tweel I, van der Graaf R, Moons KG, van Delden JJ, Reitsma JB, Koffijberg H: The need to balance merits and limitations from different disciplines when considering the stepped wedge cluster randomized trial design. *BMC Med Res Methodol* 15:93, 2015
16. Ferrara A, Hedderson MM, Albright CL, Brown SD, Ehrlich SF, Caan BJ, Sternfeld B, Gordon NP, Schmittdiel JA, Gunderson EP, Mevi AA, Tsai AL, Ching J, Crites Y, Quesenberry CP, Jr.: A pragmatic cluster randomized clinical trial of diabetes prevention strategies for women with gestational diabetes: Design and rationale of the gestational diabetes' effects on moms (gem) study. *BMC Pregnancy Childbirth* 14:21, 2014
17. Hemming K, Haines TP, Chilton PJ, Girling AJ, Lilford RJ: The stepped wedge cluster randomised trial: Rationale, design, analysis, and reporting. *BMJ* 350:h391, 2015
18. Hemming K, Lilford R, Girling AJ: Stepped-wedge cluster randomised controlled trials: A generic framework including parallel and multiple-level designs. *Stat Med* 34:181-196, 2015

Research plan- Helena Fadl
