# Supplementary material for: Changing diagnostic criteria for gestational diabetes (CDC4G) in Sweden: A stepped wedge cluster randomised trial
Source: PLoS Med. 2024 Jul 8;21(7):e1004420. doi: 10.1371/journal.pmed.1004420 (PMC11262657; doi:10.1371/journal.pmed.1004420)
Supplement: S2 Appendix — (PDF) [file pmed.1004420.s003.pdf]

Bästa resultat får du om du öppnar den här PDF-portföljen i Acrobat X eller Adobe Reader X eller senare.

Hämta Adobe Reader nu!
