# Supplementary material for: Changing diagnostic criteria for gestational diabetes (CDC4G) in Sweden: A stepped wedge cluster randomised trial
Source: PLoS Med. 2024 Jul 8;21(7):e1004420. doi: 10.1371/journal.pmed.1004420 (PMC11262657; doi:10.1371/journal.pmed.1004420)
Supplement: S1 Table — (PDF) [file pmed.1004420.s006.pdf]

S1 Table. Intention to treat population in the CDC4G study according to cluster and number of births registered at delivery unit*

Clusters	Delivery units	Intention to treat population*	Number of birthsaccording to the medical birth register
C11Gotland	Visby Hospital	413	413
C10Västerås	Västerås Central Hospital	2 666	2 666
C9 Malmö	Skåne University Hospital, Malmö	5 756	5 735
C8 Stockholm	Stockholm South General Hospital	21 672	6 202
	Danderyds Hospital		7 818
	BB Stockholm		
	Karolinska University Hospital, Huddinge		3 401
	Karolinska University Hospital, Solna		2 559
	Södertälje Hospital		1 553
C7 Halland	Varberg Hospital	2 826	1447
	Halmstad		1343
C6 Göteborg	Gothenburg Sahlgrenska University Hospital	9 305	9 302
C5 Lund	Skåne University Hospital, Lund	3 607	3 589
C4 Kristianstad	Kristianstad Central Hospital	1 940	1 916
C3 Örebro	Örebro University hospital	3 288	2 826
	Karlskoga Hospital		434
C2 Uppsala	Uppsala University Hospital	4 240	4 240
C1 Dalarna	Falun Hopital	2 670	2 670
Total**		58 383	58 114

*The intention to treat population was defined according to the study protocol, according to the oral glucose tolerance test dates, or gestational week if no OGTT done at included clusters (table S6). Numbers of pregnancies in the study are not equal to number of births per cluster, as some births may have occurred at another clinic.
