# Supplementary material for: Changing diagnostic criteria for gestational diabetes (CDC4G) in Sweden: A stepped wedge cluster randomised trial
Source: PLoS Med. 2024 Jul 8;21(7):e1004420. doi: 10.1371/journal.pmed.1004420 (PMC11262657; doi:10.1371/journal.pmed.1004420)
Supplement: S2 Table — (PDF) [file pmed.1004420.s007.pdf]

S2 Table: List of the CDC4G clusters, number of births, methods for screening and diagnosing GDM [1]

Cluster	Number of births/ year[a]	Indication for diagnostic 75 g OGTT									Diagnostic criteria prior to switch (mmol/L)	Overt diabetes[e] (mmol/L)	Method for glucose analysis	Additive in blood collection tubes
		History				Clinical indication								
		GDM	Macro-somia[b]	LGA[c]	IUFD	BMI	FH[d]	Polyhydramnios	Accel growth	RBG				
Gotland[h]	538	24-28	24-28	-	-	≥35	24-28	Yes	Yes	RPG[f]≥9.0 mmol/L at enrollment week 25, 29, 32/33, 37/38.	FPG <7.0 and 2-h PG 8.9–11.0	FPG ≥7.0 and/or 2-h PG ≥11.1	HemoCue AB HemoCue 201 RT (glucose dehydrogenase) No external control	EDTA[g]
Västerås[i]	3 120	24-28	24-28	24-28	-	≥35	-	Yess	Yes	RPG[e]≥9.0 mmol/L at booking week 25, 30, 35.	FPG <7.0 and 2-h PG 8.9–11.0	FPG ≥7.0 and/or 2-h PG ≥11.1	Beckman Coulter Au (hexokinase) Accredited method	NaF, Citrate buffer, EDTA (VACUETTE® FC Mix Tube)
Stockholm[j]	28 602	T1& 24-28	24-28	24-28	-	≥35	-	Yes	Yes	RPG[e] ≥9.0mmol/L at booking, week 25, 29, 32/33, 37/38.	FPG <7.0 and 2-h PG 8.9–11.1	FPG ≥7 .0 and/or 2-h PG ≥11.2	Roche Cobas Beckman Coulter Au Siemens Advia (hexokinase) Accredited methods	NaF, Citrate buffer, EDTA (VACUETTE® FC Mix Tube)
Halland[k]	4 446	12/ 24-28	-	24-28	24-28	≥30	24-28	Yes	Yes	RPG[e] ≥8.0 mmol/L at enrollment, week 12, 28/29, 32, 37	FPG <7.0 and 2-h PG 9.0–11.1	FPG ≥7.0 and/or 2-h PG ≥11.2	HemoCueAB HemoCue 201 RT (glucose dehydrogenase) External control (lab)	EDTA[g]
Gothenburg[l]	9 550	25-29	25-29	25-29	-	≥35	25-29	Yes	(Yes)	Within one week if RPG[e] 8.0–12.1 mmol/L at first antenatal care visit, week 25, 28–29, 35–36.	FPG≥7.0 and/or 2-h PG ≥10.0 RPG[c] ≥12.2	Not defined	Nova biomedical StatStrip TM Multi-Well ™ (glucose oxidase) External control (lab)	Li-heparin without gel[g]
Örebro[m]	3 565	T1 & 24-28	24-28	24-28	-	≥35	24-28/40	Yes	Yes-within 3 days	RPG[e] ≥9 .0mmol/L at booking, week 24, 28/29, 33, 37.	FPG <7.0 and 2-h PG 8.9–11.0	FPG ≥7.0 and/or 2-h PG ≥11.1	Siemens Advia (hexokinase) Accredited method	NaF, Citrate buffer, EDTA (VACUETTE® FC Mix Tube)
Uppsala[n]	4 200	12-14/24-28	24-28/40	24-28/40	-	≥30	24-28	Yes	Yes	RPG[e] ≥8.8 mmol/L at booking, week 25, 28/29, 33, 37	FPG ≥7.0 and/or 2-h PG ≥10.0	Not defined	Abbott Architect (hexokinase) Accredited method	NaF, Citrate buffer, EDTA (VACUETTE® FC Mix Tube)
Dalarna[o]	3 232	12-14/24-28	24-28/40	24-28/40	-	>35	24-28/40	Yes	Yes	RPG[e] ≥9.0 mmol/L at booking, week 24, 28/29, 33, 37.	FPG <7.0 and/or 2-h PG ≥8.9	FPG ≥7.0 and/or 2-h PG ≥11.1	Siemens Advia (hexokinase) Accredited method	NaF, Citrate buffer, EDTA (VACUETTE® FC Mix Tube)
Malmö[p]	4 944	10-12	10-12	-	-	≥35	10-12	Yes	No	Capillary 75g OGTT week 28 in all women. FBG[e] ≥7 and/or 2-h PG[e] ≥10.0 mmol/L indication for a diagnostic OGTT	FPG ≥7.0 and/or 2-h PG ≥9.0	Not defined	Roche Cobas (hexokinase) Accredited method	NaF, Citrate buffer, EDTA (VACUETTE® FC Mix Tube)
Lund[q]	3 703	10-12	10-12	-	-	≥35	10-12	Yes	No					
Kristianstad[r]	2 085	10-12	10-12	-	-	≥35	10-12	Yes	No					

When not otherwise stated, glucose measurement is based on venous plasma.

BMI=body mass index. FH=family history. FPG=fasting plasma glucose. IUFD=intrauterine fetal death. LGA=large for gestational age. OGTT=oral glucose tolerance test. PG=plasma glucose. RPG=random plasma glucose. SPR=Swedish Pregnancy Register. T1=trimester 1.

[a]Number of births per year based on data from the SPR 2017.

[b]Defined as birthweight ≥4 500g.

[c]Defined as birth weight ≥+2 standard deviations above the Swedish reference curve [2].
[d]In Dalarna, Malmö, Lund, Kristianstad, Uppsala, Gotland, Halland defined as first degree relative with type 1 or type 2 diabetes, otherwise first degree relative with type 2 diabetes.
[e] Diagnosed and treated as GDM but with rapid management by specialist care unit in contrast to usual maternal healthcare.
[f] Based on capillary samples.
[g] Patient-near analysis.
[h]Gotland (Visby Hospital).
[i]Västerås (Västerås Central Hospital).
[j]Stockholm (Stockholm South General Hospital, Danderyds Hospital, Karolinska University Hospital, Huddinge BB Stockholm, Karolinska University Hospital, Solna, Södertälje Hospital.)
[k]Halland (Varberg Hospital) .
[l]Gothenburg (Gothenburg Sahlgrenska University Hospital).
[m]Örebro (Örebro University hospital, Karlskoga Hospital).
[n]Uppsala (Uppsala University Hospital)
[o]Dalarna (Falun Hospital).
[p]Malmö (Skåne University Hospital, Malmö).
[q]Lund (Skåne University Hospital, Lund).
[r]Kristianstad (Kristianstad Central Hospital)

1. Fadl H. Impact on pregnancy outcomes when changing diagnostic criteria for gestational diabetes in Sweden [Internet]. ISRCTN 2017 [updated 2022 Dec 19; cited 2023 Dec 19]. Available from: https://doi.org/10.1186/ISRCTN41918550.
2. Maršál K, Persson PH, Larsen T, Lilja H, Selbing A, Sultan B. Intrauterine growth curves based on ultrasonically estimated foetal weights. Acta Paediatr. 1996;85(7):843-8.
