# Supplementary material for: Changing diagnostic criteria for gestational diabetes (CDC4G) in Sweden: A stepped wedge cluster randomised trial
Source: PLoS Med. 2024 Jul 8;21(7):e1004420. doi: 10.1371/journal.pmed.1004420 (PMC11262657; doi:10.1371/journal.pmed.1004420)
Supplement: S3 Table — (PDF) [file pmed.1004420.s008.pdf]

S3 Table. Algorithm for starting (A), titration (B), maximum dose (C) for treatment (lifestyle advice, metformin and insulin) in the CDC4G trial[1]

	Dose (mg/IU)
Lifestyle advice*	Dietitian
Metformin†	
A. Start dose	500x1
B.Titration dose	500x/3rd day
C. Maximum dose	1 000x3
Insulin‡	
A. Start dose (mmol/L)§	
Fast acting insulin. 1 hour postprandial cPG	
8–10	4
>10	6
Intermediate acting insulin. Fasting cPG	
5.3–6.0	6
> 6.0	8
B.Titration dose (mmol/L)¶	
FPG	
<4	- 2
4.0–5.3	± 0
5.3–6	+ 2
>6	+ 4
Postprandial glucose	
<6	- 2
6–8	± 0
8–10	+ 2
>10	+ 4

cPG=capillary plasma glucose. FPG=fasting plasma glucose.
*All women diagnosed with GDM were offered lifestyle advice. If ≥3 glucose values above target during 1 week led to pharmacological treatment.
†Overweight or high fasting and basal glucose levels.
‡When metformin is not expected to bring hyperglycaemia rapidly under control or considered inappropriate for clinical reasons or declined by the patient. If fasting plasma glucose above the target, intermediate acting insulin was the first line of choice. Long acting analogue insulins if the plasma glucose targets were not reached. Rapid acting insulin analogues was added when elevated postprandial glucose levels.
§Starting doses are 4–8 units depending on the glycaemia and other clinical factors (e.g. BMI).
¶Evaluation of glucose values and titration of insulin dose twice a week initially, after which titration is performed once a week. Changes in insulin doses if ≥3 glucose values above target during 1 week.

1. Fadl H. Impact on pregnancy outcomes when changing diagnostic criteria for gestational diabetes in Sweden [Internet]. ISRCTN 2017 [updated 2022 Dec 19; cited 2023 Dec 19]. Available from: https://doi.org/10.1186/ISRCTN41918550.
