# Supplementary material for: Changing diagnostic criteria for gestational diabetes (CDC4G) in Sweden: A stepped wedge cluster randomised trial
Source: PLoS Med. 2024 Jul 8;21(7):e1004420. doi: 10.1371/journal.pmed.1004420 (PMC11262657; doi:10.1371/journal.pmed.1004420)
Supplement: S4 Table — (PDF) [file pmed.1004420.s009.pdf]

S4 Table. Target plasma glucose during pharmacological treatment [1]

Timing*	Target for cPG (mmol/L)
Fasting	<5.3
Before other meals	<6
1-hour after meal	<8
Before bedtime	<7

cPG=capillary plasma glucose.
* cPG is measured 4 times/day in lifestyle treatment group and 7 times/day in pharmacological treated group. Self-measurement of plasma glucose.

1. Fadl H. Impact on pregnancy outcomes when changing diagnostic criteria for gestational diabetes in Sweden [Internet]. ISRCTN 2017 [updated 2022 Dec 19; cited 2023 Dec 19]. Available from: https://doi.org/10.1186/ISRCTN41918550.
