# Supplementary material for: Changing diagnostic criteria for gestational diabetes (CDC4G) in Sweden: A stepped wedge cluster randomised trial
Source: PLoS Med. 2024 Jul 8;21(7):e1004420. doi: 10.1371/journal.pmed.1004420 (PMC11262657; doi:10.1371/journal.pmed.1004420)
Supplement: S5 Table — (PDF) [file pmed.1004420.s010.pdf]

S5 Table. The minimal requirements on obstetric surveillance during 2018 for participating centers [1]

All	Dietary treatment	Metformin/insulin treatment
Written information about diet and exercise	If plasma glucose within goal levels no other controls except ultrasound fetal weight estimation gestational week 38 for pre-delivery assessment.	Ultrasound fetal weight estimation at least 2 times, gestational week 28-32 and latest at gestational week 38.
Conventional maternal healthcare controls	Induction of labor when indicated according to current guidelines at the clinic (provided that ultrasound fetal weight estimation is normal).	Induction of labor if not delivered, at the latest gestational week 40+6.

1. Fadl H. Impact on pregnancy outcomes when changing diagnostic criteria for gestational diabetes in Sweden [Internet]. ISRCTN 2017 [updated 2022 Dec 19; cited 2023 Dec 19]. Available from: https://doi.org/10.1186/ISRCTN41918550.
