# Supplementary material for: Changing diagnostic criteria for gestational diabetes (CDC4G) in Sweden: A stepped wedge cluster randomised trial
Source: PLoS Med. 2024 Jul 8;21(7):e1004420. doi: 10.1371/journal.pmed.1004420 (PMC11262657; doi:10.1371/journal.pmed.1004420)
Supplement: S6 Table — (PDF) [file pmed.1004420.s011.pdf]

S6 Table. Pregnancies included in the CDC4G trial based on gestational week, OGTT dates and GDM status

Pregnancies	**2017**	**Study period (2018)**	**2019**
No OGTT		No OGTT and ≥28+0 GW[†]	2018 population followed 90 days post-partum[§]
OGTT	GDM and ≤23+6 GW[*]	OGTT[‡]	

GDM=gestational diabetes mellitus. GW=gestational week. OGTT=oral glucose tolerance test.
[*]All pregnancies diagnosed with GDM during 2017, but not passed 23+6 gestational week 2018-01-01 are included in the OGTT population.
†All pregnancies without an OGTT from 28+0 gestational weeks on 2018-01-01 up to 2018-12-31 are included in the non- OGTT group.
‡All pregnancies with an OGTT during 2018-01-01—2018-12-31 are included in the OGTT group unless the indication for the OGTT is polyhydramnios, suspected LGA or OGTT ≥ 36+0 without information on indication, in which case the original group is maintained.
[§]Pregnancies included in the trial during the later study periods (months) will be followed until 90 days after birth. Last birth 4th of august 2019.
