# Supplementary material for: Changing diagnostic criteria for gestational diabetes (CDC4G) in Sweden: A stepped wedge cluster randomised trial
Source: PLoS Med. 2024 Jul 8;21(7):e1004420. doi: 10.1371/journal.pmed.1004420 (PMC11262657; doi:10.1371/journal.pmed.1004420)
Supplement: S7 Table — (PDF) [file pmed.1004420.s012.pdf]

S7 Table. Coverage and certification level for data sources used in the CDC4G trial

	Source (year of foundation)	Type of acquired data	Coverage/ certification level*
**Health data registers hosted by the Swedish National Board of Health and Welfare (SNBHW) [1]**			
National patient register	Statistics of diseases and surgical procedures grouped in patient, geographical, administrative and medical data. Includes all inpatient care and since 2001 also specialised outpatient care and day surgery. (1960)	Maternal characteristics	97-99% [1, 2]
National prescribed drug register	Contains all prescribed drugs dispensed at pharmacy from Swedish eHealth Agency. (2005)	Purchased prescription drugs	100% [1]
National medical birth register	Data on maternal characteristics, delivery and neonatal outcomes from deliveries in Sweden. (1973)	Maternal characteristics, neonatal characteristics, maternal outcomes	95-99% [1]
Cause of death register	Medical death certificate of all Swedish citizens dying in Sweden or abroad. Includes underlying cause/causes of death. Since 2012 it also includes foreign residents dying in Sweden. (1961)	Maternal and neonatal death	>99% [1]
**Swedish statistics [3]**			
Statistics Sweden	Official national statistics e.g. on income, socioeconomic conditions, educational level, and country of birth, immigration, emigration. (1686)	Maternal characteristics	>99% [4]
**National quality registers hosted by the Swedish Associations of Local Authorities and Regions [5]**			
Swedish pregnancy register	Automatic transfer from journal system (2014), manual input from maternal healthcare (1999) and direct register from CUB-examinations (2006). Data on all maternal characteristics, delivery and neonatal outcomes are recorded.	Maternal characteristics, maternal outcomes, and neonatal outcomes	98.5% [6]/ Level 1 [5]
Swedish neonatal quality register	Continuous web based register with data on all neonates admitted to neonatal care units within the firsr four weeks of life. Contains data on infant mortality, neonatal morbidity, and health at follow up. (2001)	Neonatal characteristics and neonatal outcomes.	98-99% [7, 8]/ Level 1 [5]
**Researcher-generated data**			
Electronic Case Report Form	Manual module created by the CDC4G study group for completeness of data for mother and child outcomes. (2018)	Maternal characteristics, screening, OGTT data, treatment, and admissions. Neonatal outcomes and NICU admissions.	82-93% [7]

CUB=combined ultrasound and biochemistry. NICU= neonatal intensive care units. SNBHW= Swedish National Board of Health and Welfare.
*National quality registers are certified according to four levels (1,2,3,K) every year. Level 1 is the highest level that meets the criteria for level 2,3 and K and includes coverage >85%, actively available for research, used by the participating units, validated data quality, linked with Register Utiliser Tool and meeting the FAIR-principles [5].

1. Socialstyrelsen. Socialstyrelsens register (Registers of National board of health and welfare) [Internet]. Sweden2020 [cited 2023 Apr 12]. Available from: https://www.socialstyrelsen.se/statistik-och-data/register/alla-register/.
2. Ludvigsson JF, Andersson E, Ekbom A, Feychting M, Kim J-L, Reuterwall C, et al. External review and validation of the Swedish national inpatient register. BMC Public Health. 2011;11(1):1-16.
3. Statistics Sweden. [Internet]. 2022 [updated 2023 Jan 22; cited 2023 Mar 15]. Available from: www.scb.se/en/.
4. Statistics Sweden. Kvalitetsdeklaration Befolkningsstatistik. Sweden: 2018 BE0101.
5. Nationella Kvalitetsregister (National quality registers). Level of certification. [Internet]. Sweden [updated 2023 Jan 31; cited 2022 Apr 12]. Available from: https://skr.se/kvalitetsregister/drivaregister/attsokaochredovisamedel/certifieringsnivaer.54492.html.
6. Stephansson O, Petersson K, Björk C, Conner P, Wikström AK. The Swedish Pregnancy Register–for quality of care improvement and research. Acta obstetricia et gynecologica Scandinavica. 2018;97(4):466-76.
7. MedSciNet. MedSciNet Studies [Internet]. CSAM Health AS; 2021 [cited 2023 Jan 23]. Available from: www.medscinet.com.
8. Norman M, Källén K, Wahlström E, Håkansson S, Collaboration S, Skiöld B, et al. The Swedish neonatal quality register–contents, completeness and validity. Acta Paediatr. 2019;108(8):1411-8.
