# Supplementary material for: Changing diagnostic criteria for gestational diabetes (CDC4G) in Sweden: A stepped wedge cluster randomised trial
Source: PLoS Med. 2024 Jul 8;21(7):e1004420. doi: 10.1371/journal.pmed.1004420 (PMC11262657; doi:10.1371/journal.pmed.1004420)
Supplement: S8 Table — (PDF) [file pmed.1004420.s013.pdf]

**S8 Table. Comparing baseline characteristics in pregnancies with missing vs. complete potential confounding information by study groups in the modified intention to treat population**

| | Modified intention to treat population | | | | | | | | | |
|---|---|---|---|---|---|---|---|---|---|---|
| | SWE-GDM criteria (n=22 797) | | | | | WHO-2013 criteria (n=24 383) | | | | |
| | Missing potential confounders (n=994) | | Complete potential confounders (n=21 803) | | | Missing potential confounders (n=1 146) | | Complete potential confounders (n=23 137) | | |
| **Maternal characteristics** | n | | n | | P* | n | | n | | P* |
| Age at childbirth, years | 994 | 31.3 (28-35) | 21 803 | 31.7 (28-35) | 0.29 | 1 146 | 30.8 (28-35) | 23 137 | 31.3 (28-35) | 0.026 |
| Body height at first visit, cm | 454 | 167 (163-170) | 21 625 | 166 (162-170) | 0.23 | 575 | 166 (162-170) | 22 920 | 166 (161-170) | 0.46 |
| Body weight at first visit, kg | 438 | 66 (59-74) | 21 217 | 66 (59-75) | 0.90 | 556 | 66 (59-75) | 22 568 | 66 (59-75) | 0.40 |
| BMI at first visit, kg/m$^2$ | 437 | 23.5 (22-27) | 21 154 | 23.7 (21-27) | 0.80 | 550 | 23.9 (22-27) | 22 491 | 23.9 (22-27) | 0.46 |
| Underweight (<18.5) | | 14 (3.2) | | 533 (2.5) | | | 15 (2.7) | | 605 (2.7) | |
| Normal (18.5-24.9) | | 257 (58.8) | | 12 448 (58.8) | | | 317 (57.6) | | 12 840 (57.1) | |
| Overweight (25.0-29.9) | | 107 (24.5) | | 5 351 (25.3) | | | 135 (24.5) | | 5 773 (25.7) | |
| Obesity class I (30.0-34.9) | | 44 (10.1) | | 1 995 (9.4) | | | 47 (8.6) | | 2 242 (10.0) | |
| Obesity class II (35.0-39.9) | | 10 (2.3) | | 610 (2.9) | | | 23 (4.2) | | 737 (3.3) | |
| Obesity class III (≥40.0) | | 5 (1.1) | | 217 (1.0) | | | 13 (2.4) | | 294 (1.3) | |
| Parity† | 994 | | 21 803 | | 0.018 | 1 145 | | 23 137 | | 0.76 |
| 0 | | 479 (48.2) | | 9 305 (42.7) | | | 464 (40.5) | | 9 425 (40.7) | |

| | | | | | | | | | | |
|---|---|---|---|---|---|---|---|---|---|---|
| 1 | | 341 (34.3) | | 8 196 (37.6) | | | 430 (37.5) | | 8 621 (37.3) | |
| 2 | | 123 (12.4) | | 3 034 (13.9) | | | 165 (14.4) | | 3 425 (14.8) | |
| 3 | | 32 (3.2) | | 824 (3.8) | | | 60 (5.2) | | 1 054 (4.6) | |
| ≥4 | | 19 (1.9) | | 444 (2.0) | | | 26 (2.3) | | 612 (2.6) | |
| Chronic hypertension‡ | 994 | 7 (0.7) | 21 803 | 157 (0.7) | 0.95 | 1 146 | 8 (0.7) | 23 137 | 165 (0.7) | 0.95 |
| Smoking at first visit | 7 | | 21 803 | | >0.99 | 2 | | 23 137 | | >0.99 |
| No | | 7 (100.0) | | 21 087 (96.7) | | | 2 (100.0) | | 22 307 (96.4) | |
| 1-9 cig/day | | 0 (0.0) | | 597 (2.7) | | | 0 (0.0) | | 668 (2.9) | |
| ≥10 cig/day | | 0 (0.0) | | 119 (0.6) | | | 0 (0.0) | | 162 (0.7) | |
| Swedish snuff at first visit | 938 | 67 (7.1) | 21 803 | 124 (0.6) | <0.001 | 948 | 81 (8.5) | 23 137 | 155 (0.7) | <0.001 |
| Country of birth§ | 987 | | 21 803 | | <0.001 | 1 144 | | 23 137 | | <0.001 |
| Sweden | | 782 (79.2) | | 14 810 (67.9) | | | 863 (75.4) | | 15 581 (67.3) | |
| Europe except Sweden | | 69 (7.0) | | 2 311 (10.6) | | | 109 (9.5) | | 2 350 (10.2) | |
| Middle East and North Africa | | 62 (6.3) | | 2 199 (10.1) | | | 67 (5.9) | | 2 427 (10.5) | |
| North America and Caribbean | | 4 (0.4) | | 97 (0.4) | | | 6 (0.5) | | 108 (0.5) | |
| South and Central America | | 11 (1.1) | | 292 (1.3) | | | 11 (1.0) | | 277 (1.2) | |
| Africa | | 35 (3.5) | | 1 221 (5.6) | | | 53 (4.6) | | 1 509 (6.5) | |
| South East Asia | | 5 (0.5) | | 285 (1.3) | | | 11 (1.0) | | 307 (1.3) | |
| Western Pacific | | 19 (1.9) | | 588 (2.7) | | | 24 (2.1) | | 578 (2.5) | |
| Highest education, years | 958 | | 21 024 | | <0.001 | 1 102 | | 22 410 | | 0.81 |
| <9 (school education) | | 19 (2.0) | | 659 (3.1) | | | 35 (3.2) | | 823 (3.7) | |
| 9 (school education) | | 52 (5.4) | | 1 179 (5.6) | | | 72 (6.5) | | 1 379 (6.1) | |

| | | | | | | | | | | |
|---|---|---|---|---|---|---|---|---|---|---|
| 10-11 (school education) | | 37 (3.9) | | 1 493 (7.1) | | | 72 (6.5) | | 1 616 (7.2) | |
| 12 (school education) | | 249 (26.0) | | 4 922 (23.4) | | | 298 (27.0) | | 5 682 (25.3) | |
| <3 (college/university) | | 171 (17.8) | | 3 284 (15.6) | | | 161 (14.6) | | 3 337 (14.9) | |
| ≥3 (college/university) | | 423 (44.1) | | 9 255 (44.0) | | | 450 (40.8) | | 9 296 (41.5) | |
| Doctor/licentiate degree | | 7 (0.7) | | 232 (1.1) | | | 14 (1.3) | | 277 (1.2) | |

Data are n (%) or median (IQR) unless stated otherwise.

*P is calculated with t-test for continuous variables and chi-2 test for categorical variables.
