# Supplementary material for: Changing diagnostic criteria for gestational diabetes (CDC4G) in Sweden: A stepped wedge cluster randomised trial
Source: PLoS Med. 2024 Jul 8;21(7):e1004420. doi: 10.1371/journal.pmed.1004420 (PMC11262657; doi:10.1371/journal.pmed.1004420)
Supplement: S9 Table — (PDF) [file pmed.1004420.s014.pdf]

S9 Table. Primary outcome in the modified intention to treat population and subgroup discordant for definition of GDM by period and cluster

	Modified intention to treat population					Subgroup discordant for definition of GDM *				
	**SWE-GDM criteria (n=22 797)**		**WHO-2013 criteria (n=24 283)**		**WHO-2013 vs SWE-GDM**	**SWE-GDM criteria (n=956)**		**WHO-2013 criteria (n=1 239)**		**WHO-2013 vs SWE-GDM**
					**RR (95% CI)†**					**RR (95% CI)†**
	n	LGA‡	n	LGA‡		n	LGA‡	n	LGA‡	
	22 707	2 584 (11.4)	24 209	2 790 (11.5)	0.96 (0.92-1.01)	947	273 (28.8)	1 237	278 (22.5)	0.83 (0.71-0.97)
**By period**					**RR (95% CI)§**					**RR (95% CI)§**
Jan 1 to June 30					0.96 (0.91-1.01)					0.89 (0.74-1.06)
July 1 to Dec 31					0.97 (0.90-1.03)					0.72 (0.60-0.87)
**By cluster**	n		n		**RR (95% CI)****	n		n		**RR (95% CI)****
**Dalarna,C 1**	267	39 (14.6)	2 392	365 (15.3)	1.04 (0.77-1.42)	10	2 (20.0)	167	43 (25.7)	1.29 (0.36-4.58)
**Uppsala, C2**	706	86 (12.2)	3 519	419 (11.9)	0.98 (0.79-1.21)	52	8 (15.4)	152	24 (15.8)	1.03 (0.49-2.15)
**Örebro, C3**	902	110 (12.2)	2 377	284 (11.9)	0.98 (0.80-1.20)	43	9 (20.9)	154	31 (20.1)	0.96 (0.50-1.87)
**Göteborg, C4**	3 940	424 (10.8)	5 324	566 (10.6)	0.99 (0.88-1.11)	177	43 (24.3)	307	62 (20.2)	0.83 (0.59-1.17)
**Halland, C5**	1 542	181 (11.7)	1 277	131 (10.3)	0.87 (0.71-1.08)	33	6 (18.2)	21	3 (14.3)	0.79 (0.22-2.84)
**Stockholm, C6**	13 002	1 436 (11.0)	8 599	932 (10.8)	0.98 (0.91-1.06)	541	169 (31.2)	383	97 (25.3)	0.81 (0.65-1.00)
**Västerås, C7**	2 003	260 (13.0)	653	84 (12.9)	0.99 (0.79-1.25)	83	31 (37.3)	53	18 (34.0)	0.91 (0.57-1.45)
**Gotland, C8**	345	48 (13.9)	68	9 (13.2)	0.95 (0.49-1.85)	8	5 (62.5)	0	0 (0.0)	NA

Data are n (%) unless stated otherwise.

C=cluster. CI=confidence interval. GDM gestational diabetes mellitus. LGA=large for gestational age. NA=not applicable. RR=relative risk ratio.

*The cohort of women with fasting and 2-hour plasma glucose cut off between the WHO-2013 criteria and SWE-GDM criteria (fasting plasma glucose 5.1-6.9 and/or 2-h plasma glucose 8.5-8.8/8.9/9.9 mmol/L), untreated before and treated after the switch).

†Analysed with multilevel Poisson regression, adjusted for cluster as random factor and period (January-March, April-June, July-September, October-December) as fixed factor §with interaction term for study group x period (January-June vs July-December).

‡Defined as birthweight above the 90th percentile in the Swedish reference population[1] corrected for gestational age and sex..

**Unadjusted analysis with Poisson regression within each centre.

1. Maršál K, Persson PH, Larsen T, Lilja H, Selbing A, Sultan B. Intrauterine growth curves based on ultrasonically estimated foetal weights. Acta Paediatr. 1996;85(7):843-8.
