# Supplementary material for: Changing diagnostic criteria for gestational diabetes (CDC4G) in Sweden: A stepped wedge cluster randomised trial
Source: PLoS Med. 2024 Jul 8;21(7):e1004420. doi: 10.1371/journal.pmed.1004420 (PMC11262657; doi:10.1371/journal.pmed.1004420)
Supplement: S10 Table — (PDF) [file pmed.1004420.s015.pdf]

S10 Table. Pre specified secondary neonatal outcomes in the modified intention to treat population and subgroup discordant for definition of GDM

	Modified intention to treat population				Subgroup discordant for definition of GDM[*]			
	SWE-GDM criteria (n=22 797)	WHO-2013 criteria (n=24 283)	WHO-2013 vs SWE-GDM		SWE-GDM criteria (n=956)	WHO-2013 criteria (n=1 239)	WHO-2013 vs SWE-GDM	
			Adjusted 1[†] RR (95% CI)	Adjusted 2 MI[‡] RR (95% CI)			Adjusted 1[†] RR (95% CI)	Adjusted 2 MI[‡] RR (95% CI)
5 min Apgar score <4	53 (0.23)	89 (0.37)	1.66 (1.14-2.41) P[†]=0.008	1.69 (1.14-2.49) P[‡]=0.008	1 (0.10)	5 (0.40)	NA	NA
Metabolic acidosis	116 (0.51)	109 (0.45)	0.81 (0.55-1.19) P[†]=0.29	0.82 (0.55-1.20) P[‡]=0.30	6 (0.63)	9 (0.73)	NA	NA
NICU >24 hours	1 338 (5.9)	1 661 (6.8)	1.01 (0.91-1.12) P[†]=0.86	1.01 (0.90-1.14) P[‡]=0.83	63 (6.6)	103 (8.3)	0.76 (0.43-1.35) P[†]=0.35	0.80(0.46-1.39) P[‡]=0.43
Hypoxic ischaemic encephalopathy II-III	23 (0.10)	26 (0.11)	1.07 (0.43-2.66) P[†]=0.88	NA	0 (0.0)	3 (0.24)	NA	NA
Meconium aspiration syndrome	30 (0.13)	38 (0.16)	1.35 (0.69-2.67) P[†]=0.38	NA	1 (0.10)	2 (0.16)	NA	NA
Mechanical ventilation	138 (0.61)	192 (0.79)	1.42 (1.08-1.86) P[†]=0.012	1.45 (1.07-1.96) P[‡]=0.017	4 (0.42)	6 (0.48)	NA	NA
Plasma glucose <2.6 (mmol/L)	682 (3.0)	895 (3.7)	1.12 (0.89-1.42) P[†]=0.33	1.13 (0.89-1.44) P[‡]=0.31	59 (6.2)	251 (20.3)	2.88 (1.88-4.42) P[†]<0.001	2.93 (1.96-4.38) P[‡]<0.001
Hypoglycaemia needing intravenous therapy	62 (0.27)	66 (0.27)	0.83 (0.49-1.42) P[†]=0.49	0.84 (0.51-1.38) P[‡]=0.48	7 (0.73)	15 (1.2)	1.50 (0.55-4.08) P[†]=0.43	NA

Data are n (%).

RR=relative risk ratio. CI=confidence interval. GDM=gestational diabetes mellitus. MI=multiple imputation. NA= not applicable. NICU=neonatal intensive care unit.

*The cohort of women with fasting and 2-hour plasma glucose cut off between the WHO-2013 criteria and SWE-GDM criteria (fasting plasma glucose 5.1-6.9 and/or 2-hour plasma glucose 8.5-8.8/8.9/9.9 mmol/L), untreated before and treated after the switch.

[†]Analysed with multilevel mixed model adjusted for centre as random factor and period (January-March, April-June, July-September, October-December) as fixed factor. Mixed Poisson model for binary outcomes (gives relative risk ratios as association measures), mixed multi-nominal for categorical outcomes (gives odds ratios as association measures), mixed linear model for continuous outcomes (gives mean differences as association measures), and mixed negative binomial model for count data (gives mean ratios as association measures).

[‡]Adjusted for mother's age modelled by a linear, squared, and cubic term, chronic hypertension, smoking, snuff, country of birth, and parity. Multiple imputation used for missing data on potential confounding variables.
