# Supplementary material for: Changing diagnostic criteria for gestational diabetes (CDC4G) in Sweden: A stepped wedge cluster randomised trial
Source: PLoS Med. 2024 Jul 8;21(7):e1004420. doi: 10.1371/journal.pmed.1004420 (PMC11262657; doi:10.1371/journal.pmed.1004420)
Supplement: S11 Table — (PDF) [file pmed.1004420.s016.pdf]

S11 Table. Pre-specified secondary maternal outcomes in the modified intention to treat population and subgroup discordant for definition GDM

	Modified intention to treat population				Subgroup discordant for definition of GDM*			
	**SWE-GDM Criteria (n=22 797)**	**WHO-2013 criteria (n=24 283)**	**WHO-2013 vs SWE-GDM**		**SWE-GDM criteria (n=956)**	**WHO-2013 criteria (n=1 239)**	**WHO-2013 vs SWE-GDM**	
			**Adjusted 1† RR (95% CI)**	**Adjusted 2‡ RR (95% CI)**			**Adjusted 1† RR (95% CI)**	**Adjusted 2‡ RR (95% CI)**
Induction of labour	4 587 (20.1)	4 853 (20.0)	0.96 (0.91-0.99) P†=0.042	0.96 (0.92-1.00) P‡=0.060	306 (32.0)	446 (36.0)	0.97 (0.80-1.18) P†=0.79	1.01 (0.82-1.24) P‡=0.92
Hospital stay (days) §	2.52 (1.68)	2.45 (1.83)	0.99 (0.97-1.02) P†=0.74	0.99 (0.96-1.03) P‡=0.76	2.77 (1.76)	3.06 (2.04)	1.04 (0.95-1.15) P†=0.34	1.05 (0.93-1.18) P=0.40
Breastfeeding at hospital discharge¶								
No	842 (4.4)	867 (4.6)	1.38 (1.17-1.64) P†<0.001	1.34 (1.13-1.58) P‡<0.001	39 (5.7)	58 (6.5)	2.41 (1.23-4.75) P†=0.011	2.59 (1.32-5.08) P‡=0.006
Partly	3 494 (18.2)	3 252 (17.3)	1.02 (0.93-1.13) P†=0.66	0.98 (0.89-1.08)	204 (29.7)	330 (36.8)	1.73 (1.16-2.57) P†=0.007	1.67 (1.11-2.51) P‡=0.013
Fully	14 870 (77.4)	14 630 (78.0)	Reference	Reference	444 (64.6)	509 (56.7)	Reference	Reference
Breastfeeding at hospital discharge¶ MI for outcome								
No			1.56 (1.27-1.90) P†<0.001	1.43 (1.18-1.94) P‡<0.001			2.14 (1.07-4.26) P†=0.031	2.21 (1.11-4.41) P‡=0.024
Partly			0.96 (0.85-1.08) P†=0.50	0.97 (0.86-1.09) P‡=0.59			1.35 (0.94-1.95) P†=0.11	1.33 (0.91-1.94) P‡=0.14
Fully			Reference	Reference			Reference	Reference
Self-reported health during pregnancy**								
Very good / good	11 913 (79.1)	11 711 (77.6)	Reference	Reference	411 (75.3)	470 (67.2)	Reference	Reference
Neither good nor bad	1 899 (12.6)	2 006 (13.3)	1.02 (0.91-1.15) P†=0.67	1.02 (0.91-1.15) P‡=0.67	83 (15.2)	131 (18.7)	1.34 (0.89-2.01) P†=0.16	1.36 (0.90-2.05) P‡=0.14
Bad / very bad	1 246 (8.3)	1 382 (9.1)	1.07 (0.94-1.22) P†=0.31	1.08 (0.94-1.23) P‡=0.26	52 (9.5)	98 (14.0)	1.67 (1.04-2.69) P†=0.033	1.81 (1.11-2.94) P‡=0.016
Self-reported health during pregnancy**MI for outcome								
Very good / good			Reference	Reference			Reference	Reference
Neither good nor bad			0.99 (0.86-1.14) P†=0.92	1.00 (0.87-1.15) P‡=0.98			1.25 (0.87-1.79) P†=0.23	1.25 (0.86-1.81) P‡=0.24
Bad / very bad			1.04 (0.91-1.20) P†=0.52	1.05 (0.91-1.20) P‡=0.51			1.41 (0.92-2.16) P†=0.11	1.47 (0.96-2.27) P‡=0.078
Self-reported health after pregnancy ††								
Very good / good	14 089 (91.4)	14 169 (90.5)	Reference	Reference	495 (88.2)	644 (87.9)	Reference	Reference
Neither good nor bad	952 (6.2)	1 068 (6.8)	1.02 (0.87-1.18) P†=0.85	1.01 (0.86-1.18) P‡=0.91	43 (7.7)	58 (7.9)	0.95 (0.55-1.66) P†=0.87	0.99 (0.56-1.75) P‡=0.98
Bad / very bad	368 (2.4)	412 (2.6)	0.85 (0.68-1.06) P†=0.15	0.84 (0.67-1.05) P‡=0.13	23 (4.1)	31 (4.2)	1.16 (0.57-2.34) P†=0.68	1.14 (0.56-2.33) P‡=0.71
Self-reported health after pregnancy ††MI for outcome								
Very good / good			Reference	Reference			Reference	Reference

Neither good nor bad			1.03 (0.88-1.20) P[†]=0.70	1.02 (0.88-1.20) P[‡]=0.76			0.95 (0.57-1.57) P[†]=0.83	0.99 (0.59-1.65) P=0.96
Bad / very bad			0.84 (0.65-1.07) P[†]=0.16	0.84 (0.65-1.07) P[‡]=0.16			0.98 (0.52-1.86) P[†]=0.96	0.98 (0.51-1.88) P=0.96
Satisfaction with childbirth[§§]								
Moderate / high (score 5-10)	13 311 (95.0)	12 950 (94.9)	Reference	Reference	449 (94.1)	555 (94.1)	Reference	Reference
Low (score 1-4)	699 (5.0)	699 (5.1)	1.00 (0.82-1.21) P[†]=0.98	0.99 (0.82-1.20) P[‡]=0.93	28 (5.9)	35 (5.9)	1.51 (0.82-2.77) P[†]=0.18	1.53 (0.80-2.92) P=0.20
Satisfaction with childbirth[§§] MI for outcome								
Moderate / high (score 5-10)			Reference	Reference			Reference	Reference
Low (score 1-4)			0.94 (0.78-1.13) P[†]=0.53	0.95 (0.79-1.13) P[‡]=0.56			1.07 (0.55-2.09) P[†]=0.84	1.12 (0.56-2.27) P=0.74

Data are n (%) or mean (SD).

RR=relative risk ratio. CI=confidence interval. GDM=gestational diabetes mellitus. MI= multiple imputation. NA=not applicable.

[*]The cohort of women with fasting and 2-hour plasma glucose cut off between the WHO-2013 criteria and SWE-GDM criteria (fasting plasma glucose 5.1-6.9 and/or 2-hour plasma glucose 8.5-8.8/8.9/9.9 mmol/L), untreated before and treated after the switch).

[†]Analysed with multilevel mixed model adjusted for centre as random factor and period (January-March, April-June, July-September, October-December) as fixed factor. Mixed Poisson model for binary outcomes (gives relative risk ratios for relative risk associations), mixed multi-nominal for categorical outcomes (gives odds ratios as association measures), mixed linear model for continuous outcomes (gives mean differences as association measures), and mixed negative binomial model for count data (gives mean ratios as association measures).

[‡]Adjusted for mother's age modelled by a linear, squared, and cubic term, chronic hypertension, smoking, snuff, country of birth, and parity. Multiple imputation used for missing data on potential confounding variables.

[§] From delivery to discharge. In the modified intention to treat population there were missing values for 4 women in the SWE-GDM group and for 4 in the WHO-2013 group

[¶] In the modified intention to treat population there were missing values for 3 591 (15.7%) women in the SWE-GDM group and for 5 534 (22.8%) in the WHO-2013 group. In the subgroup there were missing values for 269 (28.1%) women in the SWE-GDM group and for 342 (27.6%) in the WHO-2013 group.

[**] In the modified intention to treat population there were missing values for 7 739 (33.9%) women in the SWE-GDM group and for 9 184 (37.8%) in the WHO-2013 group. In the subgroup there were missing values for 410 (42.9) women in the SWE-GDM group and for 540 (43.6%) in the WHO-2013 group

[††] In the modified intention to treat population there were missing values for 7 388 (32.4%) women in the SWE-GDM group and for 8 634 (35.6%) in the WHO-2013 group. In the subgroup there were missing values for 395 (41.3%) women in the SWE-GDM group and for 506 (40.8%) in the WHO-2013 group.

[§§] In the modified intention to treat population there were missing values for 8 787 women (38.5%) in the SWE-GDM group and for 10 634 (43.8%) in the WHO-2013 group. In the subgroup there were missing values for 479 (50.1%) women in the SWE-GDM group and for 649 (52.4%) in the WHO-2013 group.
