# Supplementary material for: Changing diagnostic criteria for gestational diabetes (CDC4G) in Sweden: A stepped wedge cluster randomised trial
Source: PLoS Med. 2024 Jul 8;21(7):e1004420. doi: 10.1371/journal.pmed.1004420 (PMC11262657; doi:10.1371/journal.pmed.1004420)
Supplement: S12 Table — (PDF) [file pmed.1004420.s017.pdf]

S12 Table. Characteristics of the modified per protocol population and subgroup discordant for definition of GDM

	Modified per protocol population				Subgroup discordant for definition of GDM *			
	SWE-GDM criteria (n=22 757)		WHO-2013 GDM criteria (n=21 886)		SWE-GDM criteria (n=956)		WHO-2013 GDM criteria (n=1 195)	
Maternal characteristics	n		n		n		n	
Age at childbirth (years)	22 757	31.6 (28-35)	21 886	31.3 (28-35)	956	32.1 (29-36)	1 195	32.8 (29-36)
Body height at first visit (cm)	22 039	166 (162-170)	21 243	166 (161-170)	933	166 (162-170)	1 179	165 (160-170)
Body weight at first visit (kg)	21 617	66 (59-75)	20 899	66 (59-75)	922	86 (70-100)	1 161	83 (70-98)
BMI at first visit (kg/m^2)	21 553	23.7 (21.5-27.0)	20 823	23.9 (21.5-27.3)	922	30.9 (25.6-35.9)	1 159	30.5 (25.6-35.8)
Underweight (<18.5)		547 (2.5)		567 (2.7)		5 (0.5)		7 (0.6)
Normal (18.5-24.9)		12 694 (58.9)		11 996 (57.6)		192 (20.8)		247 (21.3)
Overweight (25.0 - 29.9)		5 447 (25.3)		5 274 (25.3)		212 (23.0)		286 (24.7)
Obesity class I (30.0-34.9)		2 031 (9.4)		2 045 (9.8)		211 (23.0)		265 (22.9)
Obesity class II (35.0-39.9)		614 (2.8)		677 (3.2)		219 (23.7)		248 (21.4)
Obesity class III (≥40.0)		220 (1.0)		264 (1.3)		83 (9.0)		106 (9.1)
Parity†	22 757		21 885		956		1 195	
0		9 763 (42.9)		9 411 (43.0)		304 (31.8)		360 (30.1)
1		8 528 (37.5)		8 094 (37.0)		369 (38.6)		448 (37.5)
2		3 152 (13.8)		2 965 (13.5)		180 (18.8)		221 (18.5)
3		853 (3.8)		889 (4.1)		54 (5.6)		109 (9.1)
≥4		461 (2.0)		526 (2.4)		49 (5.1)		57 (4.8)
Chronic hypertension‡	22 757	163 (0.7)	21 886	153 (0.7)	956	23 (2.4)	1 195	17 (1.4)
Smoking at first visit	21 771		20 885		927		1 163	
No		20 135 (96.4)		20 135 (96.4)		880 (94.9)		1 103 (94.8)
1-9 cig/day		595 (2.7)		600 (2.9)		37 (4.0)		49 (4.2)
≥10 cig/day		119 (0.5)		150 (0.7)		10 (1.1)		11 (0.9)
Swedish snuff at first visit	22 701	190 (0.8)	21 802	213 (1.0)	955	8 (0.8)	1 189	18 (1.5)
Country of birth§	22 750		21 885		956		1 195	
Sweden		15 569 (68.4)		14 743 (67.4)		588 (61.5)		648 (54.2)
Europe except for Sweden		2 374 (10.4)		2 257 (10.3)		98 (10.2)		127 (10.6)
Middle East and North Africa		2 253 (9.9)		2 265 (10.3)		139 (14.5)		177 (14.8)
North America and Caribbean		100 (0.4)		100 (0.5)		3 (0.3)		4 (0.3)
South and Central America		303 (1.3)		266 (1.2)		14 (1.5)		16 (1.3)
Africa		1 255 (5.5)		1 424 (6.5)		77 (8.0)		132 (11.0)
South East Asia		289 (1.3)		283 (1.3)		18 (1.9)		49 (4.1)
Western Pacific		607 (2.7)		547 (2.5)		19 (2.0)		42 (3.5)
Highest education (years)	21 946		21 190		921		1 151	
<9 (school education)		676 (3.1)		787 (3.7)		49 (5.3)		85 (7.4)
9 (school education)		1 228 (5.6)		1 313 (6.2)		65 (7.1)		102 (8.9)
10-11 (school education)		1 528 (7.0)		1 523 (7.2)		79 (8.6)		130 (11.3)
12 (school education)		5 160 (23.5)		5 339 (25.2)		248 (26.9)		298 (25.9)
<3 (college/university)		3 449 (15.7)		3 191 (15.1)		145 (15.7)		161 (14.0)
≥3 (college/university)		9 666 (44.0)		8 818 (41.6)		323 (35.1)		366 (31.8)
Doctor/licentiate degree		239 (1.1)		219 (1.0)		12 (1.3)		9 (0.8)
Plasma glucose in OGTT group (mmol/L)								

Fasting	3 085	5.0 (0.8)	3 436	5.0 (0.7)	956	5.4 (0.4)	1 195	5.5 (0.5)
1-hour	80	8.0 (2.1)	3 027	8.2 (2.0)	21	7.8 (1.4)	1 081	9.3 (1.8)
2-hour	3 040	7.1 (2.0)	3 371	7.0 (1.8)	945	7.1 (1.3)	1 168	7.9 (1.8)
HbA1c in GDM group	288	37.6 (6.6)	694	34.7 (4.9)	0	NA	597	34.4 (4.2)
Neonatal characteristics	22 698		21 838		951		1 194	
Boy		11 642 (51.3)		11 099 (50.8)		493 (51.8)		610 (51.1)
Girl		11 056 (48.7)		10 739 (49.2)		458 (48.2)		584 (48.9)

Data are n (%) or mean (SD) unless stated otherwise.

BMI=body mass index. GDM=gestational diabetes mellitus. OGTT= oral glucose tolerance test

*The cohort of women with fasting and 2-hour plasma glucose cut off between the WHO-2013 criteria and SWE-GDM criteria (fasting plasma glucose 5.1-6.9 and/or 2-hour plasma glucose 8.5-8.8/8.9/9.9) mmol/L), untreated before and treated after the switch.

†Numbers of previous deliveries; stillbirths or live births.

‡Hypertension before pregnancy or new onset hypertension with blood pressure ≥140/90 mmHg before gestational week 20.

§Grouped according to International Diabetes Federation Atlas except for having an extra category for Sweden [1].

1. International Diabetes Federation. IDF Diabetes Atlas [Internet]. 2021 [cited 2023 Apr 02]. 10th:[Available from: www.diabetesatlas.org.
