# Supplementary material for: Changing diagnostic criteria for gestational diabetes (CDC4G) in Sweden: A stepped wedge cluster randomised trial
Source: PLoS Med. 2024 Jul 8;21(7):e1004420. doi: 10.1371/journal.pmed.1004420 (PMC11262657; doi:10.1371/journal.pmed.1004420)
Supplement: S13 Table — (PDF) [file pmed.1004420.s018.pdf]

S13 Table. Primary and pre-specified neonatal outcomes in the modified per protocol population and subgroup discordant for definition of GDM

	Modified per protocol population						Subgroup discordant for definition of GDM *					
	SWE-GDM criteria (n=22 757)		WHO-2013 criteria (n=21 886)		WHO-2013 vs SWE-GDM		SWE-GDM criteria (n=956)		WHO-2013 criteria (n=1 195)		WHO-2013 vs SWE-GDM	
					Adjusted 1† RR (95% CI)	Adjusted 2 MI‡ RR (95% CI)					Adjusted 1† RR (95% CI)	Adjusted 2 MI ‡ RR (95% CI)
**Primary outcome**												
Large for gestational age (>90th percentile) [1]	22 668	2 573 (11.4)	21 820	2 496 (11.3)	0.96 (0.91-1.01)<br>P†=0.13	0.96 (0.90-1.03)<br>P‡=0.29	947	273 (28.8)	1 193	273 (22.9)	0.81 (0.69-0.94)<br>P†=0.007	0.84 (0.70-1.01)<br>P‡=0.059
**Secondary outcome**												
Composite neonatal outcome	22 757	288 (1.3)	21 996	322 (1.5)	1.11 (0.97-1.27)<br>P†=0.12	1.15 (0.98-1.35)<br>P‡=0.094	956	15 (1.6)	1 195	13 (1.1)	0.18 (0.08-0.39)<br>P†<0.001	NA
Respiratory distress	22 757	148 (0.65)	21 886	173 (0.79)	1.32 (1.04-1.69)<br>P†=0.023	1.41 (1.06-1.86)<br>P‡=0.016	956	6 (0.63)	1 195	6 (0.50)	NA	NA
Spinal cord injury	22 757	0 (0.00)	21 886	2 (0.01)	NA	NA	956	0 (0.00)	1 195	0 (0.00)	NA	NA
Peripheral nerve/ brachial plexus injury	22 757	16 (0.07)	21 886	21 (0.10)	1.18 (0.66-2.10)<br>P†=0.57	NA	956	1 (0.10)	1 195	2 (0.17)	NA	NA
Basal/depressed skull fracture	22 757	0 (0.0)	21 886	0 (0.0)	NA	NA	956	0 (0.0)	1 195	0 (0.0)	NA	NA
Clavicular fracture	22 757	31 (0.14)	21 886	34 (0.16)	0.73 (0.35-1.53)<br>P†=0.40	0.74 (0.34-1.56)<br>P‡=0.43	956	2 (0.21)	1 195	1 (0.08)	NA	NA
Long bone fracture	22 757	3 (0.01)	21 886	0 (0.00)	NA	NA	956	0 (0.0)	1 195	0 (0.0)	NA	NA
Cranial haemorrhage	22 757	37 (0.16)	21 886	50 (0.23)	1.63 (1.24-2.15)<br>P†<0.001	1.71 (1.26-2.32)<br>P‡<0.001	956	1 (0.10)	1 195	0 (0.0)	NA	NA
Stillbirth (≥22 gestational weeks) or neonatal death (day 0-28)	22 757	69 (0.30)	21 886	69 (0.32)	0.80 (0.55-1.14)<br>P†=0.22	0.79 (0.56-1.11)<br>P‡=0.17	956	5 (0.52)	1 195	1 (0.08)	NA	NA
Need of therapeutic cooling	22 757	20 (0.09)	21 886	29 (0.13)	1.33 (0.17-10.6)<br>P†=0.79	NA	956	0 (0.0)	1 195	4 (0.33)	NA	NA
Preterm birth (<37 weeks)	22 757	941 (4.1)	21 886	1 031 (4.7)	1.08 (0.95-1.21)<br>P†=0.23	1.09 (0.95-1.25)<br>P‡=0.22	956	39 (4.1)	1 195	73 (6.1)	1.19 (0.70-2.03)<br>P†=0.51	1.28 (0.72-2.27)<br>P‡=0.40
Small for gestational age (<10thpercentile) [1]	22 668	2 519 (11.1)	21 820	2 453 (11.2)	0.98 (0.91-1.05)<br>P†=0.58	0.98 (0.90-1.08)<br>P‡=0.72	947	56 (5.9)	1 193	98 (8.2)	1.12 (0.88-1.43)<br>P†=0.35	1.13 (0.81-1.57)<br>P‡=0.47
NICU >24h	22 757	1 334 (5.9)	21 886	1 538 (7.0)	1.00 (0.89-1.12)<br>P†=0.97	1.00 (0.88-1.14)<br>P‡=0.98	956	63 (6.6)	1 195	100 (8.4)	0.76 (0.42-1.37)<br>P†=0.36	0.80 (0.45-1.41)<br>P‡=0.45
Metabolic acidosis§	22 757	116 (0.51)	21 886	103 (0.47)	0.81 (0.55-1.20)<br>P†=0.30	0.82 (0.55-1.21)<br>P‡=0.31	956	6 (0.63)	1 195	9 (0.75)	NA	NA
5 min Apgar score <4	22 757	53 (0.23)	21 886	81 (0.37)	1.57 (1.06-2.32)<br>P†=0.023	1.58 (1.08-2.30)<br>P‡=0.017	956	1 (0.10)	1 195	4 (0.33)	NA	NA
Hypoxic ischaemic encephalopathy II-III	22 757	23 (0.10)	21 886	26 (0.12)	0.95 (0.40-2.25)<br>P†=0.90	NA	956	0 (0.0)	1 195	3 (0.25)	NA	NA
Meconium aspiration syndrome	22 757	29 (0.13)	21 886	35 (0.16)	1.36 (0.72-2.58)<br>P†=0.35	NA	956	1 (0.10)	1 195	2 (0.17)	NA	NA
Mechanical ventilation	22 757	138 (0.61)	21 886	164 (0.75)	1.30 (1.03-1.64)<br>P†=0.025	1.38 (1.04-1.83)<br>P‡=0.026	956	4 (0.42)	1 195	6 (0.50)	NA	NA
Plasma glucose in infants <2.6 (mmol/L)	22 757	681 (3.0)	21 886	844 (3.9)	1.11 (0.89-1.38)<br>P†=0.36	1.12 (0.89-1.41)<br>P‡=0.33	956	59 (6.2)	1 195	242 (20.2)	2.95 (1.90-4.57)<br>P†<0.001	3.00 (1.99-4.55)<br>P‡<0.001
Hypoglycaemia needing intravenous therapy	22 757	62 (0.27)	21 886	63 (0.29)	0.87 (0.53-1.41)<br>P†=0.57	0.88 (0.56-1.38)<br>P=0.58	956	7 (0.73)	1 195	15 (1.3)	1.54 (0.58-4.05)<br>P†=0.38	NA

**Exploratory outcomes**												
Macrosomia (birthweight ≥4500g)	22 668	677 (3.0)	21 820	565 (2.6)	0.79 (0.74-0.85) P†<0.001	0.79 (0.72-0.87) P‡<0.001	947	86 (9.1)	1 193	45 (3.8)	0.32 (0.19-0.53) P†<0.001	0.33 (0.20-0.54) P‡<0.001
Large for gestational age (>2 SD) [1]	22 668	1 016 (4.5)	21 820	947 (4.3)	0.89 (0.81-0.98) P†=0.018	0.90 (0.81-0.99) P‡=0.031	947	157 (16.6)	1 193	138 (11.6)	0.73 (0.53-0.99) P†=0.049	0.78 (0.63-0.98) P‡=0.032
Small for gestational age (<2 SD) [1]	22 668	658 (2.9)	21 820	699 (3.2)	1.02 (0.85-1.23) P†=0.83	1.01 (0.83-1.23) P‡=0.92	947	9 (0.95)	1 193	33 (2.8)	1.40 (0.75-2.62) P†=0.30	1.50 (0.70-3.24) P‡=0.30
Birth length (cm)	22 590	50.3 (2.4)	21 744	50.2 (2.4)	-0.08 (-0.14 to -0.02) P†=0.012	-0.06 (-0.12 to 0.01) P‡=0.10	944	50.9 (2.2)	1 191	50.3 (2.4)	-0.28 (-0.47 to -0.09) P†=0.004	-0.30 (-0.42 to -0.18) P‡<0.001
Birthweight (g)	22 668	3528 (530)	21 820	3510 (538)	-22 (-35 to -10) P†<0.001	-21 (-38 to -4) P‡=0.014	947	3 761 (552)	1 193	3 578 (560)	-144 (-193 to -95) P†<0.001	-141 (-196 to -86) P‡<0.001
Gestational age (days)	22 757	278.1 (12.1)	21 886	277.6 (12.5)	-0.44 (-0.61 to -0.28) P†<0.001	-0.43 (-0.64 to -0.22) P‡<0.001	956	277.4 (10.7)	1 195	274.8 (11.1)	-1.48 (-2.35 to -0.62) P†<0.001	-1.56 (-2.44 to -0.67) P‡<0.001

Data are n (%) or mean (SD). Effect measures as relative risk for binary variables or mean differences for continuous variables (95% CI).

CI=confidence interval. GDM=gestational diabetes mellitus. MI=multiple imputation. NA=not applicable. NICU=neonatal intensive care unit. RR=relative risk ratio. SD= standard deviation

*The cohort of women with fasting and 2-hour plasma glucose cut off between the WHO-2013 criteria and SWE-GDM criteria (fasting plasma glucose 5.1-6.9 and/or 2-hour plasma glucose 8.5-8.8/8.9/9.9 mmol/L), untreated before and treated after the switch.

†Analysed with multilevel mixed model adjusted for centre as random factor and period (January-March, April-June, July-September, October-December) as fixed factor. Mixed Poisson model for binary outcomes (gives relative risk ratios for relative risk associations), mixed multi-nominal for categorical outcomes (gives odds ratios as association measures), mixed linear model for continuous outcomes (gives mean differences as association measures), and mixed negative binomial model for count data (gives mean ratios as association measures).

‡Adjusted for mother's age modeled by a linear, squared, and cubic term, chronic hypertension, smoking, snuff, country of birth, and parity. Multiple imputation used for missing data on potential confounding variables.

§pH <7.05 and base excess >12 mmol/L in umbilical artery or pH <7.00 in umbilical artery.

1. Maršál K, Persson PH, Larsen T, Lilja H, Selbing A, Sultan B. Intrauterine growth curves based on ultrasonically estimated foetal weights. Acta Paediatr. 1996;85(7):843-8.
