# Supplementary material for: Changing diagnostic criteria for gestational diabetes (CDC4G) in Sweden: A stepped wedge cluster randomised trial
Source: PLoS Med. 2024 Jul 8;21(7):e1004420. doi: 10.1371/journal.pmed.1004420 (PMC11262657; doi:10.1371/journal.pmed.1004420)
Supplement: S14 Table — (PDF) [file pmed.1004420.s019.pdf]

S14 Table. GDM prevalence, pre-specified maternal outcomes in the modified per protocol population and subgroup discordant for definition of GDM

	Modified per protocol population						Subgroup discordant for definition of GDM*					
	SWE-GDM criteria (n=22 757)		WHO-2013 criteria (n=21 886)		WHO-2013 vs SWE-GDM		SWE-GDM criteria (n=956)		WHO-2013 criteria (n=1 195)		WHO-2013 vs SWE-GDM	
					**Adjusted 1†<br>RR (95% CI)**	**Adjusted 2 MI ‡<br>RR (95% CI)**					**Adjusted 1†<br>RR (95% CI)**	**Adjusted 2 MI ‡<br>RR (95% CI)**
**GDM prevalence**	22 757	587 (2.6)	21 886	1 485 (6.8)	2.48 (1.98-3.11)<br>P<0.001	2.45 (1.94-3.11)<br>P<0.001	956	0 (0.0)	1 195	1 195 (100)	NA	NA
**Secondary outcomes**												
Composite maternal outcome	22 757	2 709 (11.9)	21 886	2 473 (11.3)	0.95 (0.89-1.02)<br>P† =0.19	0.96 (0.91-1.02)<br>P‡=0.21	956	144 (15.1)	1 195	138 (11.5)	0.76 (0.61-0.95)<br>P† =0.018	0.78 (0.66-0.94)<br>P‡=0.007
Shoulder dystocia	22 757	59 (0.26)	21 886	38 (0.17)	0.46 (0.28-0.76)<br>P† =0.002	0.45 (0.28-0.72)<br>P‡<0.001	956	4 (0.42)	1 195	3 (0.25)	NA	NA
Perineal trauma (3rd and 4th degree)	22 757	521 (2.3)	21 886	446 (2.0)	0.78 (0.67-0.90)<br>P† <0.001	0.78 (0.67-0.90)<br>P‡<0.001	956	18 (1.9)	1 195	20 (1.7)	0.79 (0.48-1.29)<br>P† =0.34	NA
Postpartum haemorrhage (≥1000 ml)	22 757	2 263 (9.9)	21 886	2 078 (9.5)	1.00 (0.92-1.09)<br>P† =0.97	1.00 (0.93-1.08)<br>P‡=0.94	956	128 (13.4)	1 195	122 (10.2)	0.76 (0.58-0.99)<br>P† =0.042	0.79 (0.64-0.97)<br>P‡ =0.026
Treatment during pregnancy												
Diet only	22 757	264 (1.2)	21 886	822 (3.8)	3.35 (2.68-4.18)<br>P† <0.001	3.33 (2.66-4.17)<br>P‡<0.001	956	10 (1.0)	1 195	625 (52.3)	54.2 (27.6-107)<br>P† <0.001	55.4 (28.6-107)<br>P‡ <0.001
Metformin only	22 757	203 (0.89)	21 886	416 (1.9)	1.72 (1.34-2.19)<br>P† <0.001	1.67 (1.29-2.17)<br>P‡<0.001	956	0 (0.0)	1 195	345 (28.9)	NA	NA
Insulin only	22 757	37 (0.16)	21 886	75 (0.34)	1.40 (0.94-2.09)<br>P† =0.097	1.39 (0.96-2.02)<br>P‡=0.084	956	0 (0.0)	1 136	59 (4.9)	NA	NA
Metformin and insulin	21 886	188 (0.86)	22 757	109 (0.48)	1.87 (1.15-3.04)<br>P† =0.012	1.77 (1.09-2.87)<br>P‡=0.020	956	0 (0.0)	1 195	159 (13.3)	NA	NA
Gestational hypertension§	22 594	648 (2.9)	21 733	721 (3.3)	1.01 (0.72-1.40)<br>P† =0.95	1.06 (0.75-1.49)<br>P‡=0.75	933	50 (5.4)	1 178	78 (6.6)	1.27 (0.87-1.84)<br>P† 0.21	1.37 (1.01-1.86)<br>P‡ =0.046
Preeclampsia¶	22 757	564 (2.5)	21 886	612 (2.8)	1.07 (0.85-1.35)<br>P† =0.55	1.14 (0.89-1.47)<br>P‡=0.28	956	37 (3.9)	1 195	65 (5.4)	1.36 (1.01-1.84)<br>P† =0.043	1.60 (1.13-2.26)<br>P‡ =0.008
Gestational weight gain (kg)**	21 044	12.1 (5.6)	20 360	12.1 (5.8)	-0.3 (-0.4 to -0.2)<br>P† <0.001	-0.3(-0.4 to -0.2)<br>P‡<0.001	820	11.2 (6.9)	1 071	9.5 (6.6)	-1.6(-2.3 to -1.0)<br>P† <0.001	-1.5(-2.1 to -0.9)<br>P‡ <0.001
Caesarean section	22 757	4 018 (17.7)	21 886	3 837 (17.5)	1.01 (0.97-1.06)<br>P† =0.53	1.02 (0.98-1.07)<br>P‡=0.35	956	255 (26.7)	1 195	309 (25.9)	0.94 (0.79-1.11)<br>P† =0.44	0.92 (0.76-1.11)<br>P‡ =0.38
Emergency caesarean section	22 757	2 973 (13.1)	21 886	2 830 (12.9)	1.02 (0.97-1.08)<br>P† =0.37	1.02 (0.95-1.09)<br>P‡=0.60	956	187 (19.6)	1 195	212 (17.7)	0.91 (0.72-1.14)<br>P† =0.41	0.90 (0.70-1.16)<br>P‡ =0.42
Elective caesarean section	22 757	1 045 (4.6)	21 886	1 007 (4.6)	0.99 (0.92-1.05)<br>P† =0.71	0.99 (0.93-1.06)<br>P‡=0.83	956	68 (7.1)	1 195	97 (8.1)	1.06 (0.70-1.61)<br>P† =0.78	1.02 (0.65-1.60)<br>P‡ =0.91
Instrumental delivery	22 757	1 143 (5.0)	21 886	1 049 (4.8)	0.99 (0.90-1.10)<br>P† =0.86	1.00 (0.90-1.12)<br>P‡=0.94	956	31 (3.2)	1 195	43 (3.6)	1.07 (0.60-1.88)<br>P† =0.82	1.11 (0.63-1.96)<br>P‡ =0.72
Induction of labour	22 757	4 574 (20.1)	21 886	4 361 (19.9)	0.95 (0.91-1.00)<br>P† =0.055	0.96 (0.91-1.00)<br>P‡=0.056	956	306 (32.0)	1 195	433 (36.2)	0.99 (0.82-1.20)<br>P† =0.91	1.03 (0.83-1.27)<br>P‡ =0.81
Hospital stay<br>(from delivery to discharge)	22 753	2.52 (1.68)	21 883	2.49 (1.83)	1.00 (0.98-1.02)<br>P† =0.93	1.01 (0.97-1.03)<br>P‡=0.95	956	2.77 (1.76)	1 195	3.07 (2.04)	1.04 (0.95-1.14)<br>P† =0.40	1.05 (0.94-1.19)<br>P‡ =0.38

Breastfeeding at hospital discharge	19 173		18 639				687		896			
No		840 (4.4)		864 (4.6)	1.38 (1.17-1.63)<br>P† <0.001	1.33 (1.13-1.58)<br>P‡<0.001		39 (5.7)		58 (6.5)	2.42 (1.23-4.75)<br>P† =0.010	2.59 (1.32-5.08)<br>P‡ =0.006
Partly		3 485 (18.2)		3 233 (17.3)	1.02 (0.92-1.12)<br>P† =0.73	1.00 (0.91-1.11)<br>P‡=0.96		204 (29.7)		330 (36.8)	1.74 (1.17-2.58)<br>P† =0.006	1.67 (1.11-2.51)<br>P‡=0.013
Fully		14 848 (77.4)		14 542 (78.0)	Reference	Reference		444 (64.6)		508 (56.7)	Reference	Reference
Breastfeeding at hospital discharge MI												
No					1.48 (1.22-1.80)<br>P† <0.001	1.38 (1.14-1.68)<br>P‡=0.001					2.01 (1.00-4.04)<br>P† =0.049	2.08 (1.03-4.18)<br>P‡=0.041
Partly					0.97 (0.87-1.09)<br>P† =0.64	0.98 (0.87-1.09)<br>P‡=0.67					1.32 (0.90-1.94)<br>P† =0.15	1.30 (0.87-1.92)<br>P‡=0.19
Fully					Reference	Reference					Reference	Reference
Self-reported health during pregnancy††	15 030		14 215				546		679			
Very good / good		11 892 (79.1)		11 064 (77.8)	Reference	Reference		411 (75.3)		459 (67.6)	Reference	Reference
Neither good nor bad		1 898 (12.6)		1 875 (13.2)	1.04 (0.93-1.17)<br>P† =0.50	1.04 (0.93-1.17)<br>P‡=0.47		83 (15.2)		128 (18.8)	1.35 (0.90-2.03)<br>P† =0.14	1.38 (0.92-2.09)<br>P‡=0.12
Bad /very bad		1 240 (8.2)		1 276 (9.0)	1.10 (0.96-1.25)<br>P† =0.18	1.11 (0.96-1.27)<br>P‡=0.14		52 (9.5)		92 (13.5)	1.56 (0.97-2.53)<br>P† =0.069	1.69 (1.03-2.76)<br>P‡=0.037
Self-reported health during pregnancy††MI												
Very good / good					Reference	Reference					Reference	Reference
Neither good nor bad					1.01 (0.89-1.15)<br>P† =0.84	1.02 (0.89-1.16)<br>P‡=0.78					1.26 (0.87-1.81)<br>P† =0.21	1.26 (0.87-1.82)<br>P‡=0.22
Bad /very bad					1.05 (0.90-1.23)<br>P=0.49	1.06 (0.91-1.24)<br>P‡=0.44					1.35 (0.87-2.08)<br>P† =0.17	1.41 (0.90-2.19)<br>P‡=0.13
Self-reported health after pregnancy††	15 380		14 760				561		713			
Very good / good		14 064 (91.4)		13 392 (90.7)	Reference	Reference		495 (88.2)		628 (88.1)	Reference	Reference
Neither good nor bad		951 (6.2)		982 (6.6)	1.04 (0.89-1.22)<br>P† =0.59	1.04 (0.89-1.21)<br>P‡=0.64		43 (7.7)		54 (7.6)	0.91 (0.52-1.61)<br>P† =0.76	0.96 (0.54-1.70)<br>P‡=0.88
Bad /very bad		365 (2.4)		386 (2.6)	0.89 (0.71-1.11)<br>P† =0.31	0.88 (0.70-1.10)<br>P‡=0.27		23 (4.1)		31 (4.3)	1.18 (0.59-2.39)<br>P† =0.63	1.16 (0.57-2.37)<br>P‡=0.68
Self-reported health after pregnancy†† MI												
Very good / good					Reference	Reference					Reference	Reference
Neither good nor bad					1.05 (0.89-1.23)<br>P† =0.57	1.04 (0.89-1.23)<br>P‡=0.60					0.93 (0.55-1.55)<br>P† =0.77	0.96 (0.57-1.63)<br>P‡=0.89
Bad /very bad					0.86 (0.67-1.12)<br>P† =0.26	0.86 (0.67-1.12)<br>P‡=0.26					1.00 (0.53-1.89)<br>P† =0.99	1.00 (0.52-1.90)<br>P‡=0.99
Satisfaction with childbirth††	13 985		13 575				477		589			
Moderate / high (score 5-10)		13 286 (94.9)		12 880 (94.9)	Reference	Reference		449 (94.1)		554 (94.1)	Reference	Reference
Low (score 1-4)		699 (5.0)		695 (5.1)	0.99 (0.81-1.21)<br>P† =0.93	0.99 (0.82-1.19)<br>P‡=0.89		28 (5.9)		35 (5.9)	1.52 (0.83-2.79)<br>P† =0.18	1.54 (0.81-2.94)<br>P‡=0.19
Satisfaction with childbirth†† MI												

Moderate / high (score 5-10)					Reference	Reference					Reference	Reference
Low (score 1-4)					0.94 (0.79-1.13)<br>$P^{\dagger}$ =0.51	0.95 (0.80-1.13)<br>$P^{\ddagger}$=0.53					1.07 (0.55-2.06)<br>$P^{\dagger}$ =0.84	1.12 (0.56-2.23)<br>$P^{\ddagger}$=0.75

Data are n (%) or mean (SD). CI=confidence interval. GDM=gestational diabetes mellitus. MI=multiple imputation. NA=not applicable. RR=relative risk ratio.

*The cohort of women with fasting and 2-hour plasma glucose cut off between the WHO-2013 criteria and SWE-GDM criteria (fasting plasma glucose 5.1-6.9 and/or 2-hour plasma glucose 8.5-8.8/8.9/9.9 mmol/L, untreated before and treated after the switch).

[†]Analysed with multilevel mixed model adjusted for centre as random factor and period (January-March, April-June, July-September, October-December) as fixed factor. Mixed Poisson model for binary outcomes (gives relative risk ratios for relative risk associations), mixed multi-nominal for categorical outcomes (gives odds ratios as association measures), mixed linear model for continuous outcomes (gives mean differences as association measures), and mixed negative binomial model for count data (gives mean ratios as association measures).

[‡]Adjusted for mother's age modeled by a linear, squared, and cubic term, chronic hypertension, smoking, snuff, country of birth, and parity. Multiple imputation used for missing data on potential confounding variables

.[§] Blood pressure ≥140/90 mmHg, measured two times with at least 4 hour interval during pregnancy after gestational week 20.

¶Blood pressure ≥140/90 mmHg and newly onset proteinuria ≥ 300mg/24 hours after gestational week 20.

[**]Adjusted for weight at first visit.
