# Supplementary material for: Changing diagnostic criteria for gestational diabetes (CDC4G) in Sweden: A stepped wedge cluster randomised trial
Source: PLoS Med. 2024 Jul 8;21(7):e1004420. doi: 10.1371/journal.pmed.1004420 (PMC11262657; doi:10.1371/journal.pmed.1004420)
Supplement: S15 Table — (PDF) [file pmed.1004420.s020.pdf]

S15 Table. Primary outcome in the modified per protocol population and subgroup discordant for definition of GDM by period and cluster

	Per protocol population					Subgroup discordant for definition of GDM *				
	**SWE-GDM criteria (n=22 757)**		**WHO-2013 criteria (n=21 886)**		**WHO-2013 vs SWE-GDM** **RR (95% CI)†**	**SWE-GDM criteria (n=956)**		**WHO-2013 criteria (n=1 195)**		**WHO-2013 vs SWE-GDM** **RR (95% CI)†**
	n	LGA‡	n	LGA‡		n	LGA‡	n	LGA‡	
	22 668	2 573 (11.35)	21 820	2 496 (11.44)	0.96 (0.91-1.01)	947	273 (28.8)	1 193	273 (22.9)	0.81 (0.69-0.94)
By period					RR (95% CI)§					RR (95% CI)§
**Jan 1 to June 30**					0.95 (0.89-1.02)					0.85 (0.70-1.02)
**July 1 to Dec 31**					0.97 (0.91-1.04)					0.73 (0.60-0.89)
By cluster	n		n		RR (95% CI)**	n		n		RR (95% CI)**
**Dalarna,C 1**	264	38 (14.4)	2 385	363 (15.2)	1.06 (0.78-1.44)	10	2 (20.0)	167	43 (25.7)	1.29 (0.36-4.58)
**Uppsala, C2**	705	86 (12.2)	1 173	136 (11.6)	0.95 (0.74-1.22)	52	8 (15.4)	108	19 (17.6)	1.14 (0.53-2.44)
**Örebro, C3**	900	110 (12.2)	2 375	284 (12.0)	0.98 (0.79-1.20)	43	9 (20.9)	154	31 (20.1)	0.96 (0.50-1.87)
**Göteborg, C4**	3 930	423 (10.8)	5 314	563 (10.6)	0.98 (0.87-1.11)	177	43 (24..3)	307	62 (20.2)	0.83 (0.59-1.17)
**Halland, C5**	1 539	180 (11.7)	1 275	130 (10.2)	0.87 (0.70-1.08)	33	6 (18.2)	21	3 (14.3)	0.78 (0.22-2.84)
**Stockholm, C6**	12 984	1 429 (11.0)	8 578	927 (10.8)	0.98 (0.91-1.06)	541	169 (31.2)	383	97 (25.3)	0.81 (0.65-1.00)
**Västerås, C7**	2 001	259 (12.9)	652	84 (12.9)	0.99 (0.79-1.25)	83	31 (37.3)	53	18 (34.0)	0.91 (0.57-1.45)
**Gotland, C8**	345	48 (13.9)	68	9 (13.2)	0.95 (0.49-1.85)	8	5 (62.5)	0	0 (0.0)	NA

Data are n (%) unless stated otherwise.
C=cluster. CI=confidence interval. GDM gestational diabetes mellitus. LGA=large for gestational age. MI=multiple imputation. NA=not applicable. RR=relative risk ratio.
*The cohort of women with fasting and 2-hour plasma glucose cut off between the WHO-2013 criteria and SWE-GDM criteria (fasting plasma glucose 5.1-6.9 and/or 2-h plasma glucose 8.5-8.8/8.9/9.9 mmol/L), untreated before and treated after the switch).
†Analysed with multilevel Poisson regression, cluster as random factor and period (January-March, April-June, July-September, October-December) as fixed factor..
‡Defined as birthweight above the 90th percentile in the Swedish reference population[1] corrected for gestational age and sex..
§with interaction term for study group x period (January-June vs July-December)..
**Analysed with Poisson regression within each centre.

1. Maršál K, Persson PH, Larsen T, Lilja H, Selbing A, Sultan B. Intrauterine growth curves based on ultrasonically estimated foetal weights. Acta Paediatr. 1996;85(7):843-8.
