# Supplementary material for: Changing diagnostic criteria for gestational diabetes (CDC4G) in Sweden: A stepped wedge cluster randomised trial
Source: PLoS Med. 2024 Jul 8;21(7):e1004420. doi: 10.1371/journal.pmed.1004420 (PMC11262657; doi:10.1371/journal.pmed.1004420)
Supplement: S16 Table — (PDF) [file pmed.1004420.s021.pdf]

S16 Table. Characteristics of the intention to treat population

	Intention to treat population			
	SWE-GDM criteria (n=29 349)		WHO-2013 criteria (n=29 034)	
**Maternal characteristics**	n		n	
Age at childbirth, years	29 349	31.5 (28-35)	29 034	31.3 (28-35)
Body height at first visit, cm	28 464	166 (162-170)	28 153	166 (161-170)
Body weight at first visit, kg	27 896	66 (59-75)	27 682	66 (59-76)
BMI at first visit, kg/m^2	27 808	23.8 (21.5-27.2)	27 578	24.0 (21.6-27.4)
Underweight (<18.5)		716 (2.6)		724 (2.6)
Normal (18.5-24.9)		16 118 (58.0)		15 680 (56.9)
Overweight (25.0-29.9)		7 169 (25.8)		7 171 (26.0)
Obesity class I (30.0-34.9)		2 661 (9.6)		2 734 (9.9)
Obesity class II (35.0-39.9)		849 (3.0)		907 (3.3)
Obesity class III (≥40.0)		295 (1.1)		362 (1.3)
Parity[†]	29 342		29 022	
0		12 559 (42.8)		11 889 (41.0)
1		10 960 (37.4)		10 839 (37.3)
2		4 136 (14.1)		4 256 (14.7)
3		1 096 (3.7)		1 298 (4.5)
≥4		591 (2.0)		740 (2.6)
Chronic hypertension[‡]	29 349	207 (0.7)	29 034	195 (0.7)
Smoking at first visit	28 053		27 634	
No		27 026 (96.3)		26 553 (96.1)
1-9 cig/day		858 (3.1)		866 (3.1)
≥10 cig/day		169 (0.6)		215 (0.8)
Swedish snuff at first visit	29 286	224 (0.8)	28 825	254 (0.9)
Country of birth[§]	29 339		29 032	
Sweden		19 850 (67.7)		19 625 (67.6)
Europe except Sweden		3 332 (11.4)		3 111 (10.7)
Middle East and North Africa		3 213 (10.9)		3 097 (10.7)
North America and Caribbean		125 (0.4)		132 (0.4)
South and Central America		354 (1.2)		325 (1.1)
Africa		1 392 (4.7)		1 693 (5.8)
South East Asia		337 (1.1)		341 (1.2)
Western Pacific		736 (2.5)		708 (2.4)

Highest education, years	28 289		28 111	
<9 (school education)		915 (3.2)		1 012 (3.6)
9 (school education)		1 665 (5.9)		1 763 (6.3)
10-11 (school education)		1 950 (6.9)		2 000 (7.1)
12 (school education)		6 723 (23.8)		7 222 (25.7)
<3 (college/university)		4 419 (15.6)		4 171 (14.8)
≥3 (college/university)		12 291 (43.4)		11 594 (41.2)
Doctor/licentiate degree		326 (1.1)		349 (1.2)
Plasma glucose in OGTT group, mmol/L, mean (SD)				
Fasting	8 869	4.9 (0.7)	7 746	4.8 (0.7)
1-hour	80	8.0 (2.1)	3 042	8.2 (2.0)
2-hour	9 100	6.9 (1.6)	7 808	6.8 (1.6)
HbA1c in GDM group, mean (SD)	494	36.9 (6.2)	825	34.9 (5.1)
**Neonatal characteristics**	29 289		28 977	
Boy		15 010 (51.2)		14 747 (50.9)
Girl		14 279 (48.8)		14 230 (49.1)

Data are n (%) or median (IQR) unless stated otherwise.
BMI=body mass index. GDM=gestational diabetes mellitus.NA= not applicable. OGTT=oral glucose tolerance test. SD=standard deviation
[†]Number of previous deliveries; stillbirths or live births.
[‡]Hypertension known before pregnancy or new onset hypertension with blood pressure ≥140/90 mmHg before gestational week 20.
[§]Gr
