# Supplementary material for: Changing diagnostic criteria for gestational diabetes (CDC4G) in Sweden: A stepped wedge cluster randomised trial
Source: PLoS Med. 2024 Jul 8;21(7):e1004420. doi: 10.1371/journal.pmed.1004420 (PMC11262657; doi:10.1371/journal.pmed.1004420)
Supplement: S17 Table — (PDF) [file pmed.1004420.s022.pdf]

S17 Table. Primary outcome and pre-specified neonatal outcomes in the intention to treat population

	**Intention to treat population**			
	**SWE-GDM criteria (n=29 349)**	**WHO-2013 criteria (n=29 034)**	**WHO-2013 vs SWE-GDM**	
			**Adjusted 1† RR (95% CI)**	**Adjusted 2‡ RR (95% CI)**
**Primary outcome**				
Large for gestational age (>90th percentile)§[1]	3 407 (11.7)	3 382 (11.7)	1.00 (0.93-1.08) P†=0.99	1.00 (0.93-1.08) P‡=0.92
**Secondary neonatal outcome**				
Composite neonatal outcome	365 (1.2)	447 (1.5)	1.11 (0.95-1.31) P†=0.19	1.12 (0.96-1.31) P‡=0.15
Respiratory distress	183 (0.62)	250 (0.86)	1.39 (1.10-1.75) P†=0.005	1.41 (1.11-1.78) P‡=0.004
Spinal cord injury	1 (0.00)	2 (0.01)	NA	NA
Peripheral nerve / brachial plexus injury	21 (0.07)	24 (0.08)	1.16 (0.71-1.90) P†=0.54	NA
Basal/depressed skull fracture	0 (0.0)	0 (0.0)	NA	NA
Clavicular fracture	40 (0.14)	37 (0.13)	0.69 (0.34-1.39) P†=0.30	0.71 (0.34-1.47) P‡=0.36
Long bone fracture	5 (0.02)	0 (0.0)	NA	NA
Cranial haemorrhage	48 (0.16)	64 (0.22)	1.52 (1.10-2.11) P†=0.011	1.58 (1.14-2.20) P‡=0.006
Stillbirth (≥22 gestational weeks) or neonatal death (day 0-28)	96 (0.33)	115 (0.40)	0.83 (0.61-1.12) P†=0.23	0.84 (0.61-1.15) P‡=0.27
Need of therapeutic cooling	25 (0.09)	31 (0.11)	1.24 (0.48-3.21) P†=0.65	NA
Preterm birth (<37 weeks)	1 275 (4.3)	1 397 (4.8)	1.02 (0.93-1.12) P†=0.61	1.03 (0.93-1.15) P‡=0.55
Small for gestational age (<10th percentile)§ [1]	3 185 (10.9)	3 220 (11.1)	0.97 (0.91-1.04) P†=0.43	0.98 (0.91-1.05) P‡=0.53
**Exploratory neonatal outcomes**				
Macrosomia (birthweight ≥4500g)§	862 (2.9)	785 (2.7)	0.81 (0.71-0.93) P†=0.002	0.83 (0.72-0.95) P‡=0.008
Large for gestational age (>2 SD) §[1]	1 345 (4.6)	1 281 (4.4)	0.93 (0.84-1.02) P†=0.11	0.93 (0.85-1.02) P‡=0.14

Small for gestational age (<2 SD) § [1]	828 (2.8)	919 (3.2)	1.01 (0.84-1.21)<br>P†=0.89	1.01 (0.83-1.22)<br>P‡=0.93
Birthweight (g)§	3 528 (531)	3 513 (546)	-15 (-28 to -79)<br>P†=0.038	-14 (-30 to 2)<br>P‡=0.088
Birth length (cm)¶	50.2 (2.4)	50.2 (2.4)	-0.06 (-0.11 to -0.01)<br>P†=0.012	-0.05 (-0.10 to -0.00)<br>P‡=0.046
Gestational age (days)	278.0 (12.2)	277.6 (12.9)	-0.34 (-0.57 to -0.11)<br>P†=0.003	-0.34 (-0.58 to -0.10)<br>P‡=0.006

Data are n (%) or mean (SD). Effect measures as relative risk for binary variables or mean differences for continuous variables (95% CI). RR=relative risk ratio. CI=confidence interval. GDM=gestational diabetes mellitus. MI=multiple imputation. NA= not applicable. NICU=neonatal intensive care unit.
†Analysed with multilevel mixed model adjusted for centre as random factor and period (January-March, April-June, July-September, October-December) as fixed factor. Mixed Poisson model for binary outcomes (gives relative risk ratios as association measures), mixed multi-nominal for categorical outcomes (gives odds ratios as association measures), mixed linear model for continuous outcomes (gives mean differences as association measures), and mixed negative binomial model for count data (gives mean ratios as association measures).
‡Adjusted for mother's age modelled by a linear, squared, and cubic term, chronic hypertension, smoking, snuff, country of birth, and parity. Multiple imputation used for missing data on potential confounding variables.
§In the intention to treat population there were missing values for 122 neonates in the SWE-GDM group and for 106 in the WHO-2013 group.
¶ In the intention to treat population there were missing values for 311 neonates in the SWE-GDM group and for 272 in the WHO-2013 group.

1. Maršál K, Persson PH, Larsen T, Lilja H, Selbing A, Sultan B. Intrauterine growth curves based on ultrasonically estimated foetal weights. Acta Paediatr. 1996;85(7):843-8.
