# Supplementary material for: Changing diagnostic criteria for gestational diabetes (CDC4G) in Sweden: A stepped wedge cluster randomised trial
Source: PLoS Med. 2024 Jul 8;21(7):e1004420. doi: 10.1371/journal.pmed.1004420 (PMC11262657; doi:10.1371/journal.pmed.1004420)
Supplement: S18 Table — (PDF) [file pmed.1004420.s023.pdf]

S18 Table. Pre-specified maternal outcomes in the intention to treat

	Intention to treat population			
	SWE-GDM criteria (n=29 349)	WHO-2013 criteria (n=29 034)	WHO-2013 vs SWE-GDM	
			Adjusted 1† RR (95% CI)	Adjusted 2‡ RR (95% CI)
Composite maternal outcome	3 327 (11.3)	3 184 (11.0)	0.96 (0.90-1.02) P†=0.20	0.97 (0.92-1.02) P‡=0.25
Shoulder dystocia	92 (0.31)	51 (0.18)	0.40 (0.26-0.63) P†<0.001	0.40 (0.26-0.61) P‡<0.001
Perineal trauma (III-IV)	650 (2.2)	602 (2.1)	0.84 (0.73-0.97) P†=0.016	0.85 (0.73-0.99) P‡=0.045
Postpartum haemorrhage (≥1000 ml)	2 756 (9.4)	2 655 (9.1)	1.00 (0.91-1.08) P†=0.92	1.00 (0.93-1.08) P‡=0.92
Treatment during pregnancy				
Diet only	393 (1.3)	940 (3.2)	2.99 (1.84-4.86) P†<0.001	2.98 (1.84-4.82) P‡<0.001
Metformin only	272 (0.9)	483 (1.7)	1.92 (1.33-2.76) P†<0.001	1.89 (1.31-2.71) P‡<0.001
Insulin only	62 (0.21)	88 (0.30)	1.23 (0.85-1.78) P†=0.27	1.22 (0.85-1.75) P‡=0.27
Metformin and insulin	132 (0.45)	216 (0.74)	1.94 (1.15-3.27) P†=0.013	1.88 (1.11-3.14) P‡=0.019
Gestational hypertension§	784 (2.7)	890 (3.1)	1.00 (0.75-1.33) P†=0.99	1.02 (0.76-1.38) P‡=0.87

Preeclampsia¶	770 (2.6)	816 (2.8)	1.04 (0.87-1.25)<br>P†=0.63	1.10 (0.88-1.37)<br>P‡=0.40
Gestational weight gain (kg)**	12.2 (5.7)	12.2 (5.8)	-0.2 (-0.4 to 0.02)<br>P†=0.070	-0.2 (-0.4 to 0.1)<br>P‡=0.15
Caesarean section	5 060 (17.2)	5 000 (17.2)	0.99 (0.94-1.04)<br>P†=0.64	1.00 (0.96-1.05)<br>P‡=0.95
Emergency caesarean section	3 737 (12.7)	3 636 (12.5)	0.98 (0.91-1.06)<br>P†=0.70	0.99 (0.93-1.06)<br>P‡=0.82
Elective caesarean section	1 323 (4.5)	1 364 (4.7)	0.99 (0.89-1.11)<br>P†=0.90	1.00 (0.90-1.12)<br>P‡=0.96
Instrumental delivery	1 573 (5.4)	1 519 (5.2)	0.99 (0.92-1.08)<br>P†=0.89	1.00 (0.92-1.09)<br>P‡=0.91

Data are n (%) or mean (SD).
RR=relative risk ratio. CI=confidence interval. GDM=gestational diabetes mellitus. MI= multiple imputation. NA=not applicable.
†Analysed with multilevel mixed model adjusted for centre as random factor and period (January-March, April-June, July-September, October-December) as fixed factor. Mixed Poisson model for binary outcomes (gives relative risk ratios as association measures), mixed multi-nominal for categorical outcomes (gives odds ratios as association measures), mixed linear model for continuous outcomes (gives mean differences as association measures), and mixed negative binomial model for count data (gives mean ratios as association measures).
‡Adjusted for mother's age modelled by a linear, squared, and cubic term, chronic hypertension, smoking, snuff, country of birth, and parity. Multiple imputation used for missing data on potential confounding variables.
§Blood pressure ≥140/90 mmHg, measured two times with at least 4-hour interval after gestational week 20.
Women with chronic hypertension diagnosis were excluded.
¶Blood pressure ≥140/90 mmHg and newly onset proteinuria ≥300 mg/24 hours after gestational week 20.
**Adjusted for weight at first visit. In the modified intention to treat population there were missing values for 2 162 women in the SWE-GDM group and for 2 445 in the WHO-2013 group.
