# Supplementary material for: Changing diagnostic criteria for gestational diabetes (CDC4G) in Sweden: A stepped wedge cluster randomised trial
Source: PLoS Med. 2024 Jul 8;21(7):e1004420. doi: 10.1371/journal.pmed.1004420 (PMC11262657; doi:10.1371/journal.pmed.1004420)
Supplement: S19 Table — (PDF) [file pmed.1004420.s024.pdf]

S19 Table. List of site principal investigators

Centre	Name
Gotland	Åsa Hedqvist
Västerås	Anette Gunnarsson
Stockholm	Elisabeth Storck-Lindholm and Sophia Brismar Wendel
Halland	Ann Johansson
Gothenburg	Verena Sengpiel
Örebro	Helena Backman
Uppsala	Annika Esscher
Dalarna	Sara Hogmark
Malmö	Kerstin Berntorp and Karl Kristensen
Lund	Helena Strevens
Kristianstad	Helene Holmer
