# Supplementary material for: Changing diagnostic criteria for gestational diabetes (CDC4G) in Sweden: A stepped wedge cluster randomised trial
Source: PLoS Med. 2024 Jul 8;21(7):e1004420. doi: 10.1371/journal.pmed.1004420 (PMC11262657; doi:10.1371/journal.pmed.1004420)
Supplement: Attachment — Submitted filename: Response to reviewers R2.docx [file pmed.1004420.s025.docx]

**Response to the reviewers and editors**

While scrutinising the data used in the previous analysis, we noted that a minority of pregnancies in one cluster had been included erroneously due to a coding error, and I apologise for this mistake. These pregnancies have now been removed from the analysis (this did not influence the results notably). We have now thoroughly double-checked all data and reconducted all original analysis, as well as undertaking the new analysis suggested by the reviewers and editors. The new results do not alter any of the main conclusions and have only resulted in small non-significant changes in some estimates and confidence intervals.

In our response below we refer to page and line numbers in the clean version of the manuscript and supplemental appendix.

**Requests from the editors**

**In order for the paper to be considered further at PLOS Medicine, it will need to comply with PLOS' data policy** (<https://eur02.safelinks.protection.outlook.com/?url=https%3A%2F%2Fjournals.plos.org%2Fplosmedicine%2Fs%2Fdata-availability&data=05%7C02%7Chelena.backman%40regionorebrolan.se%7C1efc12bc20424d56896a08dc186d3640%7Caece5b1982274c2782181aea120ec062%7C0%7C0%7C638412104546340332%7CUnknown%7CTWFpbGZsb3d8eyJWIjoiMC4wLjAwMDAiLCJQIjoiV2luMzIiLCJBTiI6Ik1haWwiLCJXVCI6Mn0%3D%7C3000%7C%7C%7C&sdata=J%2BlsBA85NMX%2BEZnwWZr0Vyil5TX6P90%2BeaN%2B0412Ejw%3D&reserved=0>): i.e., **anonymised study data should be made available in a publicly accessible repository, or a non-author contact provided for inquiries about access to the data.**

*Response:*

*Thank you; we have provided a non-author contact:* [*foudatauttag@regionorebrolan.se*](mailto:foudatauttag@regionorebrolan.se) *for inquiries about access to data and changed the text on page 39:*

*To the following:*

*”The datasets generated and/or analysed during the current study are not publicly available due to current Swedish ethical legislation and European union GDPR act, but are available from the organisation on reasonable request, if appropriate permits are obtained from adequate authorities. Requests should be directed to: ”* [*foudatauttag@regionorebrolan.se*](mailto:foudatauttag@regionorebrolan.se)*”.*

**Our academic editor noted that a full ITT analysis should be presented alongside the mITT analysis.**

**Where available, please quote p values alongside 95% CI.**

*Response:*

*Thank you, we have presented ITT results in the supplementary tables S16-18, and reported p-values in tables mITT tables 2-3.*

**Noting comments from the referees, please provide the full trial protocol as an attachment, along with a statistical analysis plan.**

*Response:*

*Thank you, we have uploaded the trial protocol included in the original ethical application and SAP with corrections*.

*** Reviewer #1:

The authors conducted a national stepped-wedge cluster RCT to determine whether the switch to the WHO-2023 diagnostic criteria in Sweden improves neonatal outcomes. The modified ITT analysis was conducted for eight clusters (14 delivery centres). The GDM prevalence increased after the switch to the WHO-2023 diagnostic criteria, though there was no change in the primary outcome of LGA. The subgroup analysis among women discordant between the two diagnostic criteria for GDM suggests the switch to the WHO-2023 criteria improved neonatal outcomes, including the statistically non-significant reduction of ~ 14% in the primary outcome of LGA (aRR: 0.86; 95% CI: 0.74 to 1.01).

The study was well-designed and executed; while the analysis was appropriately conducted. The manuscript is well written. Nevertheless, the authors might wish to address a couple of issues in the subsequent submission.

Major issues:

1. The Abstract should be revised as the Conclusions that implementing the WHO-2023 diagnostic criteria "decreased several adverse maternal and neonatal outcomes" were not supported by the findings.

The analysis indicates the improved neonatal outcomes were found only in the subgroup of women discordant in the GDM diagnostic criteria, but not in the whole study population. Importantly, the switch to the WHO-2023 diagnostic criteria was associated with a significantly increased risk of several predefined neonatal outcomes (i.e., cranial haemorrhage, respiratory distress and 5-m Apgar score<4). The reduced risk of several exploratory neonatal outcomes (i.e., mean birthweight, birth length, mean gestational age, macrosomia and LGA 2SD) associated with the intervention should be interpreted with caution given their exploratory nature.

*Response:*

*Thank you for this comment, we agree with the reviewer.*

*We have clarified the conclusion with the following sentence:*

***” Implementing the WHO-2013 criteria in Sweden did not significantly reduce LGA prevalence, in the total population, but statistically and clinically significantly decreased adverse composite maternal and neonatal outcomes in the subgroup discordant for the definition of GDM. Future studies are needed to evaluate the effects of treating different glucose tresholds during pregnancy in different populations, with different screening strategies and clinical management guidelines, to optimize women´s and children´s health in the short and long term.”***

*As for the increased adverse outcomes seen in the mITT population: (i.e., cranial haemorrhage, respiratory distress, mechanical ventilation and 5-min Apgar score<4) we have commented in the discussion section regarding our interpretation since we had no diagnoses of cranial hemorrhage and few with respiratory distress in the subgroup actually affected by treatment: both of these diagnoses were part of the composite outcome. For the Apgar-related outcome, there were also too few in the subgroup to give an accurate estimate; and therefore the interpretation is that the intervention is very unlikely to contribute to the result in the mITT population. This is the reason why we have not emphasized this result in the abstract, but reported it in results and discussed the matter in the discussion. We hope that this adequately addresses this point.*

*We have also clarified the following sentence in the discussion page 33, lines 533-538:*

***“In the mITT population, there were adverse neonatal outcomes (respiratory distress, mechanical ventilation and cranial hemorrhage) that are unlikely to be a result of implementation of the WHO-2013 criteria since the sample size of the subgroup discordant for definition of GDM is small (only ~4% of the study population) and very few or no adverse outcomes were seen in the subgroup affected by the intervention.”***

2. Management of missing data should be described in detail.

-	The authors might wish to add a sentence or two to describe whether a complete-case approach was conducted for missing data on the outcomes of interest.

Given substantial magnitude of missing data on breastfeeding at discharge, an exploratory analysis using multiple imputation should be considered to its related discussion stronger.

*Response:*

*Thank you. We have undertaken multiple imputation for the breastfeeding and self-reported health outcomes as a sensitivity analysis, shown in supplemental table S11. Given the degree of missingness, we have not emphasised these results in the paper. We have also reported baseline characteristics in pregnancies with missing vs complete potential confounding information by study groups, by adding a table in supplemental appendix (S8) and referring to it in results section, page 30 line 505 to 509.*

It is reasonable to conduct multiple imputation for missing covariates. Please describe the magnitude and pattern of missing data on covariates. Importantly, given 10 imputed data sets generated for the missing potential confounding variables, the authors might wish to describe how the model parameters were calculated from these imputed data sets.

*Response:*

*Thank you.*

*We report results for both complete and imputed data ands the magnitude of missingness is generally low, imputation seems to be a reasonable approach. We have added information in the results section, page :* ***“In the SWE-GDM group there were 994 (4.4%) pregnancies with missing information on potential confounders and 1146 (4.7%) in the WHO-2013 group, as reported in table S8..”***

*Also; in methods section, the following text was added on page 16, line 367-371:*

***“The STATA command mi estimate was used to adjust for variability between the ten imputed data sets according to the combination rules of Rubin [21]. Due to large number of missing outcome data for breastfeeding at discharge, self-reported health during and after pregnancy, multiple imputation was also used for these outcomes as sensitivity analysis.”***

3. The unexpected increase in several predefined neonatal outcomes associated with the switch to the WHO-2023 diagnostic criteria is worth further discussion as the explanation "these findings were not seen in the subgroup" might be driven by small size of this subgroup (~ 4% of the study population in the primary analysis).

*Response:*

*Thank you, we agree. We have expanded the discussion on this and changed this sentence on page 33 to:*

***“******In the mITT population, there were adverse neonatal outcomes (respiratory distress, mechanical ventilation and cranial hemorrhage) that are unlikely to be a result of implementation of the WHO-2013 criteria since the sample size of the subgroup discordant for definition of GDM is small (only ~4% of the study population) and very few or no adverse outcomes were seen in the subgroup affected by the intervention.”***

Minor issues:

1. There were 11 clusters created from 17 delivery units, and a cluster randomisation was stratified by centre size. Details of delivery units, including their size by specified clusters should be described and presented, probably as a supplementary table.

*Response:*

*Thank you; the number of births in the eight clusters that are included in the mITT population is reported in table S2. We have added a separate supplemental table S1 with all 17 units (ITT with excluded clusters) and number of births in each. The Stockholm cluster contains six delivery units and had to be considered as a single cluster since patients move beween units and delivery wards during pregnancy and are not just under one unit´s care. We have added the following text in the methods section, page 9 line 193-196:*

***“Delivery units in Stockholm were considered as one cluster since guidelines; surveillance, diagnosis, and treatment of GDM were uniform and women might change caregivers (midwifes and delivery units) during pregnancy. Therefore, a women could not be identified as belonging to just one delivery unit, as described in table S1”***

2. Please complete this sentence "The switch was associated with p self reported health during pregnancy (good/very good), breastfeeding at discharge in the subgroup however 17.7-36.7% of the pregnancies were missing respectively (table S10)."

*Response:*

*Thank you for noticing this mistake, and we also noted that the percentages were not correct if calculating missing values on the whole population for these variables. After requested MI for outcome analysis, the significance for self reported health disappeared.*

*There is variation for self-reported health, depending on timing before, during, or after pregnancy. We have changed the sentence to the following in resultss, p 32 line 505-509:*

***“The switch was associated with reduced breastfeeding at discharge in the subgroup discordant for definition of GDM, which was also seen in the sensitivity analysis after multiple imputation of missing outcome data. The significant increase in poorer self-reported health during pregnancy, disappeared after imputation (table S11).”***

*** Reviewer #2:

Statistical review

This paper reports a stepped wedge trial evaluating how the introduction of WHO-2013 criteria for diagnosing gestational diabetes affected maternal and neonatal outcomes.

The statistical methods used are appropriate and results are generally reported clearly. I had some comments on the reporting of the study:

1. Abstract: "After implementation of uniform clinical and laboratory guidelines, clusters were randomised to intervention (switch to WHO-2013 GDM criteria) each month from February to November 2018." - I would change to 'a number of clusters were randomised' or something similar to ensure it's clear that switching of clusters was during different months.

*Response:*

*Thank you for this response, we agree.*

*We have changed the wording according to your suggestion:*

***“After implementation of uniform clinical and laboratory guidelines, the clusters were randomised to intervention (switch to WHO-2013 GDM criteria) each month from February to November 2018”***

2. Abstract: adding a brief description of mITT definition would be useful.

*Response:*

*Thank you, we have added a sentence to describe the mITT population as follows:*

***“Primary analysis was by modified intention to treat (mITT), excluding three clusters that were randomised before study start but were unable to implement the intervention.”***

3. Abstract: for the primary outcome I feel it's appropriate to include a p-value as the trial was powered to test the associated hypothesis.

*Response:*

*Thank you; we have added p-values for outcomes in abstract and in tables 2 and 3.*

4. Abstract: could it be made clearer whether the subgroup was pre-specified?

*Response:*

*Thank you; we have clarified this aspect in the abstract:* ***”pre-specified subgroup analysis was undertaken among those discordant for the definition ofGDM.”***

5. Abstract - "There were risk reductions": I would add 'significant.'

*Response:*

*Thank you;*

*We have changed this sentence to:*

***“There were statistically significant risk reductions in the subgroup for the composite neonatal (15/956 (1.6%) versus 13/1239 (1.0%); aRR 0.19 (95% CI 0.09-0.40, p<0.001)) and composite maternal (144/956 (15.1%) vs 141/1239 (11.4%); aRR 0.78 (95% CI 0.64-0.94, p=0.009)) outcomes.”***

6. Abstract - "Implementing the WHO-2013 criteria in Sweden did not reduce the main": I would add 'significantly'. In the conclusion it is not clear that the 'decreased serious adverse … outcomes' is in the subgroup. Coming back to this after reading the paper, it might be referring to components of the composite outcome, which might be worth clarifying in the results of the abstract.

*Response:*

*Thank you, we agree that it is somewhat unclear. We have added ”significantly” following your suggestion; and also clarified that the the risk reduction is the reduction for the composite neonatal and maternal outcome:*

***”There were statistically significant risk reductions in the subgroup for the composite neonatal (15/956 (1.6%) versus 13/1239 (1.0%); aRR 0.19 (95% CI 0.09-0.40, p<0.001)) and composite maternal (144/956 (15.1%) vs 141/1239 (11.4%); aRR 0.78 (95% CI 0.64-0.94, p=0.009)) outcomes.”***

*And for the conclusion we have changed to the following:*

***“ Implementing the WHO-2013 criteria in Sweden did not significantly reduce LGA prevalence, in the total population, but statistically and clinically significantly decreased adverse composite maternal and neonatal outcomes in the subgroup discordant for the definition of GDM.”***

7. Page 7 "Pre-defined exclusion criteria according to SAP" - I did not follow how these could be pre-defined in a document published after the trial completed recruitment. I think a protocol would be needed to be included.

*Response:*

*Thank you for this comment. The study protocol will be included in this revision with the SAP.*

*This study is different to RCT designs requiring individual consent, with individual data collected as part of the trial, so some aspects of the methods differ from a classical RCT design. All pregnancies in clinical care were included in data collection at all centers, and data from medical records were transferred automatically to both quality registers (online data for delivery units) as well as to the Medical Birth Register, which is mandatory for all births in Sweden. We obtained the data from these registers after data were recorded for all births between 2017-2019: these data were delivered to us by the National Board of Health and Welfare.*

*No exclusions or inclusions for the study were performed during the data collection period of the trial. No informed consent from patients was required as they received standard healthcare during a period when guidelines were changing. The clinical guidelines and implementation of the intervention were carefully planned and monitored.*

*Exclusion of pregnancies was therefore only performed at the analysis stage and after the SAP was finalized. Choice of variables for the analysis and information about the quality of the register data was assessed throughout the study, prior to completion of the SAP to ensure the study was conducted in the most appropriate way.*

*It should be emphasized that changes in use of variables did not occur after the SAP was completed and analysis begun. The main outcomes were specified before the trial began and never altered. Examples of improvements made prior to finalizing the SAP and analysis include the addition of needing therapeutic cooling to identify severe asphyxia as this has become a national recommendation and therefore an important measure.*

*Some of the changes made after publication of the study protocol and prior to completion of the SAP were due to changes in data quality in the registers, with variations over the years. Prior to finalizing the SAP, a Core Outcome Set (COS) paper on GDM was published (1) and the recommendations from that paper were influential in identifying some secondary and exploratory variables to ensure that our study added further information based on the COS. The SAP was signed and all decisions about secondary and exploratory outcomes were made before analysis of any type was performed.*

*Therefore, “pre-specified” means “pre-specified by the SAP”, before analysis was performed on these extensive register data.*

*To clarify for readers, editors, and reviewers we have added additional information in the methods section on “Data collection” with a description of the process in this study, that is different from the usual RCT procedures:*

**”Data collection**

***Data were collected on all pregnancies in clinical care at the included clusters in the study between 2017 and 2019 using national registers and electronic case report forms. Data quality is described in supplementary table S7 [9, 12]. No exclusions or inclusions were made at study start or during the data collection phase. Health and quality registers provided standardised medical information on all pregnancies, with a coverage of >95% [15]. Pregnancy outcome data from the Swedish pregnancy quality register is available online for all clinics for health care quality surveillance. The Swedish National Board of Health and Welfare completed merging of data using personal identification numbers after data collection was completed. This was done after their review of ethical permission and according to Swedish laws and regulations. The final pseudonymised merged dataset containing data on pregnancies from 2017-2019 was received in 2022 (delayed due to the Covid pandemic). The study cohort and pre-defined outcomes for analysis are described in the SAP published in October 2022 (supplemental information). Exclusion of pregnancies was only performed at the analysis stage and after the SAP was finalized. Choice of variables for the analysis and information about quality of the register data was assessed throughout the study, prior to completion of the SAP to ensure the study was conducted in the most appropriate way.***

***No statistical analysis was performed before the SAP was published.”***

8. Page 9: it wasn't too clear to me how the randomisation was stratified by centre size - is this Gothenburg and Stockholm being treated separately or something in addition to this?

*Response:*

*Thank you, we have tried to clarify this by adding the following text in the methods section page 11, line 235-240:*

***“A cluster randomisation of allocation ratio of 1:1 stratified by centre size in two strata by size was conducted. The two largest trial clusters (Gothenburg and Stockholm) were paired in one stratum and randomised to change GDM criteria in June and August of 2018, respectively. The second stratum comprised the remaining nine clusters randomised to the intervention (WHO-2013 criteria), one cluster each month (period), from February to July and September to November 2018 (figure 1).”***

9. When I was invited to review the paper I was advised by the editor that they had some concerns about changes between the protocol and what was actually done, especially the change in analysis population and the addition of outcomes. They suggested a section that specifically mentions these changes and adds rationale for each - I would agree that this is important to add so the reader can judge how this affects the results.

*Response:*

*Thank you for this comment. In the text added on ”data collection” in the methods section, we have tried to explain the procedures in Sweden when studies are based on data from national registers including ethical amendments done and changes of some variables. We considered it very important to emphasize that no analysis was performed before the SAP was signed and published, and tried to clarify this in the methods section; page 12.*

10. I would recommend that results for all 11 clusters are presented as supplementary, although agree that the mITT analysis is likely the more robust analysis.

*Response:*

*Thank you, we have performed the ITT analysis and the results are presented in supplementary tables S16-S18.*

11. Table 2 and 3: I would personally recommend adding p-values which I feel add additional information to the confidence intervals and the overall conclusion of whether results were statistically significant (which I assume refers to p<0.05).

*Response:*

*Thank you, we have added p-values to the tables 2 and 3 for the mITT, and in supplemental S10-11, S13-14, S17-18., and in abstract and results.*

James Wason

*** Reviewer #3:

Review for Plos Medicine: Changing diagnostic criteria for gestational diabetes (CDC4G) in Sweden: A stepped wedge cluster randomised trial, de Brun M et al.

Thank you very much for asking me to review this well written manuscript. I would like to start by applauding the authors and the whole trial team for undertaking this enormous task. In this large step wedge cluster randomised trial investigating the effect of implementation of WHO-2013 GDM criteria, the authors conclude that changing criteria did not impact the primary outcome (LGA) although did improve a number of secondary outcomes, particularly amongst a subgroup discordant for diagnostic criteria.

Specific comments (mainly clarification):

P8: Second paragraph - 'The primary analysis' … through to 'due to protocol violation' - Can you please go through this paragraph to clarify the concepts - it is not clear to me as is written:

For example, the sentence commencing 'The intention to treat population….' can you please define briefly (perhaps in brackets) which eligibility criteria needed to be fulfilled before intervention to enable inclusion in the mITT as opposed to ITT?

*Response:*

*Thank you; we agree it is not clearly described. The SAP with description of eligibility criteria will be added as supplemental information with this revision. We have trie to clarify with the following sentence, page 10, line 211:*

***“The primary analysis was performed according to the intention-to-treat principle, consisting of all eligible pregnancies in the randomised clusters. The intention to treat population defined in the SAP describes the modified intention to treat (mITT) population and includes only the centres that fulfilled the eligibility criteria (introducing venous OGTT for diagnosis of GDM) before intervention [14]”***

In addition, the following sentence commencing 'The same analyses were performed in a pre-specified subgroup of women', through to the end of the brackets, is unclear - please clarify.

I think it would also help if the subgroup was 'labelled' here for the first time, and throughout the text for example as you have labelled in the table - 'Subgroup discordant for diagnosis of GDM'.

*Response:*

*Thank you for this comment. We have clarified the sentence according to your suggestion, page 10, line 216:*

*”* ***The same analyses were performed in a pre-specified subgroup of pregnancies that were untreated before randomisation and treated after the switch based on the fasting and 2-hour plasma glucose cut off values; i.e. discordant for definition of GDM.”***

Can you also emphasise here the detail that the 1hr glucose was not included for this analysis (and why).

*Response*:

*Thank you, we have clarified this as follows page 10, line 220:*

***“One-hour values were not recorded before the switch due to laboratory and clinical regulatory issues making it impossible to blind the one-hour value in clinical practice for patients and caregivers.”***

P9 in the 'Procedures' section. Could you please define specifically here what the screening method to decide who will undergo OGTT diagnostic testing is in Sweden ('The screening method remained unchanged').

*Response:*

*Thank you for this comment, we have clarified which tables report the different screening factors for triaging of performing an OGTT. All centers used random blood glucose measurements 4-6 times during pregnancy as a screening factor for triaging to performing an OGTT alongside risk factors such as: high BMI, previous GDM, previous macrosomic or LGA baby, previous stillbirth, accelerated growth, family history of diabetes. Supplementary table S2 describes screening guidelines in each center.*

P12 - Secondary maternal outcomes: Please add in a little detail about which tools were used to assess self-reported health during, and after pregnancy, and satisfaction with childbirth.

*Response:*

*Thank you, we have added a reference for the self-reported health instrument used in the Pregnancy Register and clarified that the scale used is from 1-10 for satisfaction with child birth, page 15, line 330:*

***“…self-reported health during and after pregnancy (very good ,good, neither good nor bad, bad, very bad), and satisfaction with childbirth measured at discharge (1 indicates worst experience and 10 best experience) [18]”***

P14 - 3rd paragraph of Results: In the text it isn't clear who the comparator groups are - presume from table higher BMI, parity etc is WHO 2013 vs SWE-GDM but not specified. Equally, when discussing the subgroup women, presume from table this is WHO-2013 vs SWE-GDM, but please specify in the text. Please also define 'the subgroup' here (e.g., as suggested above) and at the start of the second paragraph P15.

*Response:*

*Thank you, we have clarified the comparison group,page 18, line 398 :*

***“the women in the WHO-2013 criteria group had higher BMI and parity, were more likely to smoke, were less likely to be Swedish-born and had a lower education level compared to the SWE-GDM group (table 1).”***

*And*

***“Women in the subgroup discordant for definition of GDM were younger…”***

P15 - final sentence is unclear - what is 'p self-reported health during pregnancy' - think this might be a typo? And does the 17.7% refer to the questionnaire, and the 36.7% to breastfeeding info? Please clarify.

*Response:*

*Thank you, we apologize for this mistake. MI analysis for outcomes was requested by another reviere, and this result has changed.The sentence is corrected as follows, page 32, line 505:*

***“The switch was associated with reduced breastfeeding at discharge in the subgroup discordant for definition of GDM, which was also seen in the sensitivity analysis after multiple imputation of missing outcome data. The significant increase in poorer self-reported health during pregnancy, disappeared after imputation (table S11).***

P17 - Comparison with other studies: This paragraph could perhaps be made more granular, including a discussion of the similarities as well as defining the differences in outcomes.

*Response:*

*Thank you, we have expanded the discussion with comparisons with the GEMS study results.*

P18 - Please clarify (correct?) the first sentence here - 'routines for supplementary feeding may partly explain this difference'.

*Response:*

*Thank you, we have clarified the sentence that we believe explains this comment in a better way, page 35, line 569:*

***“..there were differences in breastfeeding rates at discharge between the studies: routines for supplementary feeding [27] might partly explain this difference. For example, in New Zealand Dextrose gel was fully implemented during the period when the GEMS study was conducted[28] In Sweden Dextrose gel was recommended in the national guidelines for the first time in 2017[29] Thus, not fully implemented during the CDC4G study. In addition, given the high proportion of missing values for self-reported breastfeeding in our study, these results should be treated with some caution”.***

End P18/beginning P19 - with the exclusions, was the trial still adequately powered?

*Response:*

*Thank you, based on the power calculation the study is adequately powered (details in method part page 15) even though three centers were excluded. We have added a comment on this in the disussion /strengths page 36, line 595****:***

***”*** ***Even though three clusters were excluded from analysis, the study had adequate statistical power. ”***

P19/20 - Implications for practice and future research: I don't come away from reading this with a sense of what the authors think is the best approach going forward based on the results of this study and other evidence, perhaps being more of a list than a discussion? Might it be possible to rewrite a little?

For example, I wonder whether you feel able yet to comment as to whether Sweden plans to continue with the newly formed process or revert or what next steps will be?

*Response:*

*Thank you, we have tried to explore in a more clear way what we believe are the next steps for Sweden. The National Board of Health and Welfare is reviewing their recommendation from 2015 on diagnosis of GDM, and they will probably make a statement on screening recommendations. However, we do not know what chenages will be made in Sweden yet. The following is added to the discussion page 38:*

***“We hope that this study, together with the other major RCTs and new scientific evidence, will contribute to the process that the Swedish National Board of Health and Welfare and other professional bodies in Sweden are working on to make changes in both screening, definition, and treatment of hyperglycemia during pregnancy.”***

Is there a thought process as to what is the best approach for women who fall in the subgroup should reversion be a choice? (you might want to refer to the ongoing Dutch study precisely examining this subgroup - Tango DM, NTR7473)?

*Thank you; at the moment there is not a national recommendation but discussions amongst professionals and researchers are ongoing both within and beyond Sweden. We do not know what the outcome of these discussions will be, and think speculations about the best approach for the subgroup is beyond the scope of this paper, particularly with the new evidence around screening for and treating GDM in early pregnancy. However we agree a statement is needed across all RCTs and have added as mentioned above the following in the discussion:*

***“******We hope that this study, together with the other major RCTs and new scientific evidence, will contribute to the process that the Swedish National Board of Health and Welfare and other professional bodies in Sweden are working on to make changes in both screening, definition, and treatment of hyperglycemia during pregnancy.”***

Will it be feasible to follow up offspring (and mothers) of this cohort using Swedish registries?

*Thank you, yes. An ethical application is being prepared, and a five-year follow up of the cohort will be performed as soon as funding is obtained and permission is granted.*

Can you be more precise in the abstract and again in this section with regards to reviewing current processes?

*Response:*

*Thank you for this relevant comment. However, the word count in the abstract is already over the recommended limit, so we find it difficult to comment further in the abstract.*

*We have expanded the discussion as described above about processes (as we interpret your comment) in the discussion*: page 36.

Am I correct in thinking that you are suggesting moving away from the recommendation of using 1 'global' approach - were you perhaps thinking of a precision approach?

Thoughts on how/what?

*Response:*

*Thank you, yes at this point we do not think it is realistic to have a single global approach, although there may be a global framework. However, we hope that this study, which shows how clinical register data can be used for evaluating IRL change in routines, can be one way of assessing clinical practice and consequenses in different populations with different underlying risks and approaches to care. However the availability of such register data may be limited in some countries.*

Are you suggesting an RCT of universal 1 step 75g OGTT in that final sentence prior to the conclusion?

Given results thus far - should we? (and will anyone fund it!?)?

*Response:*

*Thank you for this question. This comment is to remind readers that we still do not have an RCT on universal one-step OGTT at 24-28 weeks’ gestation, but neither do we suggest it. The question of early screening and treatment, precision medicine, new technology, populations differences, and other factors makes it difficult to clearly state what would be the best next step. We believe that precision medicine in this field is the right way to go, but to progress this concept we need more population based data and studies in different settings as well as data on follow up studies of mother and child. Clearly a major influence on possible outcomes is the screening strategy that is adopted. While universal screening is comprehensive, the resource use implications in some settings may be prohibitive.*

In addition, the lack of cost data also feels like a limitation, and I wonder whether there should be at least some comment within this manuscript - think very important factor with arguably not too dissimilar results between groups on a population level...

*Response:*

*Thank you, in the discussion we have stated the following:*

*“The economic consequences have been assessed in conjunction with the CDC4G trial and will be reported separately” page 36, line 617.*

*A cost analysis has been performed, and a manuscript has been prepared. Cost-effectiveness modelling is currently being planned. Since the work is extensive and will be a separate paper, we have not added the results in this paper.*

P20 - 'suggesting that treatment was actually commenced at a threshold below HAPO cut-offs' - Does the evidence not suggest that using citrate tubes equates to the process that was used when collecting samples for the HAPO study - so should they not pretty much equate to 'HAPO cut-offs' as defined in the trial?

Perhaps more accurate to say that treatment would have been commenced at lower thresholds than would have been achieved should conventional NaF tubes had been used to measure glucose levels?

*Response:*

*Thank you; We have recently published a comparison between using citrate and NaF tubes (2) and found that glucose concentrations were systematically higher by approximately 0.1 mmol/l using citrate than NaF. Therefore many women being diagnosed with GDM in CDC4G likely had diagnostic glucose results below that which would have been reported using the HAPO approach. The importance of pre-analytical loss is well known.. While lifestyle treatment would have been commenced at a lower glucose threshold in these women, pharmacological treatment would have been commenced at the same thresholds as this was based upon self blood glucose monitoring results.*

*We have therefore changed the text page 37, line 633 to:*

***“suggesting that treatment was actually commenced at a threshold below the HAPO cut-offs ”***

Grammatical/other comments:

P6: International Association of the Diabetes and Pregnancy *Trial* Group's diagnostic criteria should read "International Association of Diabetes and Pregnancy Study Groups criteria"

*Response:*

*Thank you for noticing this; we have corrected the sentence as stated above.*

P15: middle first paragraph - 'after the switch but a decrease*d* in exploratory outcomes (incorrect 'd')

*Response:*

*Thank you, sentence corrected.*

P20: 'since a raised risk for SGA was reported with treatment of lower levels *(please define this - e.g., IADPSG)* of hyperglycaemia.

*Response:*

*Thank you, we have clarified in the sentence on hyperglycemia levels associated with risk increase for SGA, page 37, line 640:*

***“since a raised risk for SGA was reported with treatment of lower levels of hyperglycaemia (those treated between cut-offs for OR 1.75 and OR 2.0 from the HAPO study) [41] in early pregnancy in the TOBOGM study)”***

Last sentence before conclusion - this sentence should commence with something like 'Thus far or To date, no RCT has ….' rather than 'Today'.

*Response:*

*Thank you, we have changed the sentence to begin ”To date”.*

References:

1.	Egan AM, Bogdanet D, Griffin TP, Kgosidialwa O, Cervar-Zivkovic M, Dempsey E, et al. A core outcome set for studies of gestational diabetes mellitus prevention and treatment. Diabetologia. 2020;63(6):1120-7.

2.	Backman HE, Karefylakis C, Schwarcz E, Magnuson A, Branzell I, Nolan CJ, et al. Diagnosis of Gestational Diabetes Mellitus: How Should We Measure Glucose? Diabetes Care. 2024.
