# Supplementary material for: Changing diagnostic criteria for gestational diabetes (CDC4G) in Sweden: A stepped wedge cluster randomised trial
Source: PLoS Med. 2024 Jul 8;21(7):e1004420. doi: 10.1371/journal.pmed.1004420 (PMC11262657; doi:10.1371/journal.pmed.1004420)
Supplement: Attachment — Submitted filename: Response to editors and reviewers (2).docx [file pmed.1004420.s026.docx]

Response to Editors and reviewers on the manuscript

*Changing diagnostic criteria for gestational diabetes (CDC4G) in Sweden through a stepped wedge cluster randomised trial" (PMEDICINE-D-23-03364R2).*

Please review your reference list to ensure that it is complete and correct. If you have cited papers that have been retracted, please include the rationale for doing so in the manuscript text, or remove these references and replace them with relevant current references. Any changes to the reference list should be mentioned in the rebuttal letter that accompanies your revised manuscript.

Please note, when your manuscript is accepted, an uncorrected proof of your manuscript will be published online ahead of the final version, unless you've already opted out via the online submission form. If, for any reason, you do not want an earlier version of your manuscript published online or are unsure if you have already indicated as such, please let the journal staff know immediately at [plosmedicine@plos.org](mailto:plosmedicine@plos.org).

------------------------------------------------------------

Requests from Editors:

General comments:

Please ensure that the full trial protocol is provided as a supplementary file, and please refer to this document in the main manuscript.

**Response:**

***Thank you; we have added the original project plan in ethics application, study protocol paper as supplementary files as requested and referred to it in manuscript in the methods part line 23-234.***

Please define all abbreviations at first use.

**Response:**

***Thank you, we have checked abbreviations and believe all abbreviations are defined at first use.***

In RCTs, there is usually a distinction in the language in terms of causal vs associational for primary and secondary trial outcomes. Please use associational language for secondary outcomes.

**Response:**

***Thank you, we have changes wording according to your comments in places where we describe associations; mainly in the discussion part; shown in the tracked changes version.***

Throughout the paper, please adapt reference call-outs to the following style: "... every year [1,2]." (noting the absence of spaces within the square brackets).

**Response:**

***Thank you; the reference list is updated according to PLOS Medicine style.***

To help us extend the reach of your research, please provide any Twitter handle(s) that would be appropriate to tag, including your own, your coauthors’, your institution, funder, or lab.

**Response:**

***Örebro University, or I personally do not have an X (Twitter account), but two co-author´s have provided the following accounts:***

***@Clin_Epi***

***@DavidSi3518966***

Data availability:

Please confirm that the email address provided ([foudatauttag@regionorebrolan.se](mailto:foudatauttag@regionorebrolan.se)) is not associated with any of the study authors.

**Response:**

***The email is not associated with any authors of the study. The email is the Region Örebro County’s functional email for all research requests in Örebro Region.***

Title: Please revise your title according to PLOS Medicine's style. Please place the study design in the subtitle (ie, after a colon). We suggest “Changing diagnostic criteria for gestational diabetes (CDC4G) in Sweden: A stepped wedge cluster randomised trial” or similar

**Response:**

***Thank you,***

***We have changed the title as you suggest to “Changing diagnostic criteria for gestational diabetes (CDC4G) in Sweden: a stepped wedge cluster randomised trial”***

Abstract:

Please report your abstract according to CONSORT for abstracts (<https://eur02.safelinks.protection.outlook.com/?url=https%3A%2F%2Fwww.equator-network.org%2Freporting-guidelines%2Fconsort-abstracts&data=05%7C02%7Chelena.backman%40regionorebrolan.se%7Cd0543685f40f42a8dde008dc736bf7ac%7Caece5b1982274c2782181aea120ec062%7C0%7C0%7C638512154825647266%7CUnknown%7CTWFpbGZsb3d8eyJWIjoiMC4wLjAwMDAiLCJQIjoiV2luMzIiLCJBTiI6Ik1haWwiLCJXVCI6Mn0%3D%7C0%7C%7C%7C&sdata=1eaQ%2BeACZi94TKUWrV9Ive%2FZQhpdb8y6xBrsFCzGczs%3D&reserved=0>), following the PLOS Medicine abstract structure (Background, Methods and Findings, Conclusions).

**Response:**

***Thank you, we have followed the abstract format.***

Abstract Methods and Findings:

Please ensure that all numbers presented in the abstract are present and identical to numbers presented in the main manuscript text.

**Response:**

***Thank you, we have made the suggested changes and added results in the manuscript but deleted them from the abstract:***

***Line 487 added numbers to main text: “******There was a statistically significant decrease in the neonatal composite outcome (15/956 (1.6%) compared to 13/1239 (1.0%); aRR 0.19 (95% CI 0.09-0.40, p<0.001)).”***

***As well as, Line  521 added “******There was also a statistically significant decreased association with the composite maternal outcomes in the subgroup (144/956 (15.1%) compared to 141/1239 (11.4%) with aRR 0.78 (95% CI 0.64-0.94, p=0.009)).”***

Selected secondary outcomes should not be reported in the Abstract.

Since not all secondary/pre-specified outcomes from this RCT are reported in this manuscript, please remove results relating to selected secondary and other pre-specified outcomes from the Abstract.

**Response:**

***Thank you; we have deleted results of selected secondary outcomes from the abstract and only report the main outcome.***

Please include the important dependent variables that are adjusted for in the analyses.

**Response:**

***Thank you, we have added dependent outcome in abstract text:***

***“Multilevel mixed regression models were used to compare the outcome, LGA, between the WHO-2013 and SWE-GDM groups adjusted for…”***

In the last sentence of the Abstract Methods and Findings section, please describe 2-3 of the main limitations of the study's methodology.

**Response:**

***Thank you, we have added: “Potential limitations of this trial are mainly due to the trial design, including failure to adhere to guidelines within and between the clusters and influences of unidentified temporal variations”***

Please include the study protocol document and analysis plan, with any amendments, as Supporting Information to be published with the manuscript if accepted.

**Response:**

***Thank you; we have added the ethics project plan, study protocol paper, Statistical analysis plan with corrections as supporting files, and referred to in text methods part.***

Abstract Conclusions:

Please begin your Abstract Conclusions with "In this study, we observed ..." or similar, to summarize the main findings from your study, without overstating your conclusions. Please emphasize what is new and address the implications of your study, being careful to avoid assertions of primacy.

**Response:**

***Thank you, we have changed the Conclusions to the following:***

***”In this study, implementing the WHO-2013 criteria in Sweden with risk factor based screening, did not significantly reduce LGA prevalence defined as birthweight >90th percentile in the total population or in the subgroup discordant for the definition of GDM”.***

Author Summary:

The Author Summary should immediately follow the Abstract in your revised manuscript. Please see our author guidelines for more information: <https://eur02.safelinks.protection.outlook.com/?url=https%3A%2F%2Fjournals.plos.org%2Fplosmedicine%2Fs%2Frevising-your-manuscript%23loc-author-summary&data=05%7C02%7Chelena.backman%40regionorebrolan.se%7Cd0543685f40f42a8dde008dc736bf7ac%7Caece5b1982274c2782181aea120ec062%7C0%7C0%7C638512154825647266%7CUnknown%7CTWFpbGZsb3d8eyJWIjoiMC4wLjAwMDAiLCJQIjoiV2luMzIiLCJBTiI6Ik1haWwiLCJXVCI6Mn0%3D%7C0%7C%7C%7C&sdata=6l8T9x9kQ2hl3vclSKc%2BVPdfB%2FWvKAH1whrFYD102vo%3D&reserved=0>

Please revise “higher glycaemic to the lower glycaemic” for clarity, referring perhaps to ‘plasma glucose’ instead

Please define RCT at first use within the Author Summary.

**Response:**

***Thank you; we have changed the wording according to your suggestion and defined RCT in author summary: “randomised controlled trial (RCT)”***

Please revise “analysis was of both in a modified intention to treat (mITT) population (all pregnancies) as well as in a subgroup actually effected by the switch” for clarity - we suggest “analysis was conducted for all pregnancies, as well as in a subgroup actually affected by the switch”

**Response:**

***Thank you, we have changed the wording to: “analysis was conducted in all pregnancies (modified intention to treat (mITT)) as well as in a subgroup affected by the switch”.***

Please define LGA at first use within the Author Summary.

**Response:**

***Thank you, changed to: large for gestational age (LGA)***

Please remove all results relating to selected secondary outcomes; please only report results relating to the primary outcome (LGA).

**Response:**

*Thank you, we have revised author summary and do not report other results but have commented on outcome reporting in “What do these findings mean” and hope this could be accepted.*

In the final bullet point of ‘What Do These Findings Mean?’, please describe the main limitations of the study in non-technical language.

**Response:**

***Thank you, we have added the following: ”Limitations of this trial are mainly due to the trial design, including failure to adhere to guidelines within and between the clusters and influences of unidentified temporal variations.”***

Methods:

Please ensure that the full trial protocol is provided as a supplementary file, and refer to this early in the Methods section.

**Response:**

***Thank you; We have referred to the original project plan; added it as supplementary file; changes in text in methods as follows:*** ***This study is reported as per the Consolidated Standards of Reporting Trials extension for SW-CRT. The details of the trial methodology are described in study protocol paper well as in published SAP with corrections.***

Any changes in your analysis - including those made in response to peer review comments - should be identified as such in the Methods section of the paper, with rationale.

**Response:**

***Thank you; only changes in analysis (after SAP) were those requested by the*** ***reviewers/editors (full ITT analysis).***

***Besides the clarifications made in last revision; we have added the following at the beginning of the Methods part: “The details of the trial methodology are described in original ethics projectplan (S3 appendix), study protocol paper well as in published SAP with corrections (S2 Appendix) [9].”***

***Also from Line 343:***

***”All reported outcomes were pre-defined according to the SAP which was completed before analysis [13]. Some changes were made to the secondary outcome measures after their original description in ethics application (S20). Final decisions on outcomes were decided based on either the study protocol paper, core outcome set publication for GDM [17] and/or outcomes reported in major studies in the research field for comparison reasons. Not all outcomes in the SAP are included in this first paper but will be included in further papers.”***

***We have added the following after the description of mITT Line 264:***

***“The results for complete ITT population was calculated, which included all clusters across Sweden who agreed to participate in the CDC4G trial.”***

Please include the completed CONSORT checklist as Supporting Information. Please add the following statement, or similar, to the Methods: "This study is reported as per the Consolidated Standards of Reporting Trials (CONSORT) extension for the stepped wedge cluster randomised trial (SW-CRT) (S1 Checklist)." The guideline can be found here: <https://eur02.safelinks.protection.outlook.com/?url=https%3A%2F%2Fwww.equator-network.org%2Freporting-guidelines%2Fconsort-cluster%2F&data=05%7C02%7Chelena.backman%40regionorebrolan.se%7Cd0543685f40f42a8dde008dc736bf7ac%7Caece5b1982274c2782181aea120ec062%7C0%7C0%7C638512154825647266%7CUnknown%7CTWFpbGZsb3d8eyJWIjoiMC4wLjAwMDAiLCJQIjoiV2luMzIiLCJBTiI6Ik1haWwiLCJXVCI6Mn0%3D%7C0%7C%7C%7C&sdata=GviPpEPONJfgYECjDQ%2FhHGnRIFiP6qrfDwyMxtfvFSU%3D&reserved=0>

When completing the checklist, please use section and paragraph numbers, rather than page numbers which will likely no longer correspond to the appropriate sections after copy-editing.

**Response:**

***Thank you; we have uploaded the revised checklist according to your instructions.***

Line 185 - please define SW-CRT at first use Line 212 - The SAP only refers to ITT; please define the difference between ITT and mITT in your analyses.

**Response:**

***Thank you; SW-CRT is defined and mentioned in “introduction” , (no change done).***

***Regarding description of ITT in SAP; this is explained but clarified further, line 261 : “The intention to treat (ITT) population defined in the SAP is labelled as the modified intention to treat (mITT) population in this manuscript and includes only the centres that fulfilled the eligibility criteria (introducing venous OGTT for diagnosis of GDM).***

If results are presented for outcomes which were not prespecified in the SAP, please indicate that they were post hoc and explain why they were added. Post hoc comparisons should be presented as hypothesis generating rather than conclusive.

**Response:**

***Thank you; There are no outcomes presented which were not pre-specified in the SAP including the revised SAP. The revision to the SAP occurred before the outcome analysis (no post Hoc analysis reported).***

***On line 343, we have further tried to clarify this matter by the following text:***

***“******All reported outcomes were pre-defined according to the SAP which was completed before analysis [13]. Some changes were made to the secondary outcome measures after their original description in ethics application (S20). Final decisions on outcomes were decided based on either the study protocol paper, core outcome set publication for GDM [17] and/or outcomes reported in major studies in the research field for comparison reasons. Not all outcomes in the SAP are included in this first paper but will be included in further papers. ”***

Results:

Line 391 - please define mPP at first use.

**Response:**

***Thank you, mPP is defined earlier in study design and participants, line 273. No changes made.***

Line 429 - Please indicate which factors are adjusted for, as table S9 only provides RR values.

**Response:**

***Thank you; we have clarified regarding adjustments in Table S9, in footnotes, and checked correct wording in other tables as well.***

Discussion:

Please remove all subheadings within your Discussion e.g.

**Response:**

***Thank you, we have deleted subheadings.***

Comparisons with other studies Lines 528, 658 - please revise to reflect more associational language for secondary outcomes.

**Response:**

***Thank you, agree. We have*** ***changed wordings, in results; discussion and conclusion (seen as marked changes).***

***Similar wordings for other associations can be seen in track changes version.***

***In discussion changes made on line 597: “a substantial reduction in associations with both neonatal and maternal composite outcome” and similar wordings on lines 599, 604, 607, 629, 694, 727.***

***In results changes made on:***

***line 490, 521, 524, 527, 562, 568, 574***

Lines 596, 633, 647 - please temper assertions of primacy by adding ‘to the best of our knowledge’ or similar.

**Response:**

***Thank you; we have changed the wording to your suggestion in text .***

Please remove the information on competing interests, funding and data sharing from the end of the main text. In the event of publication, this information will appear in the article metadata, via entries in the submission form.

**Response:**

***Thank you; we have removed all the above data from the main manuscript text.***

Tables:

Please ensure to define all abbreviations used in each table in the respective table legend.

**Response:**

***Thank you; we have checked and believe we have done so according to your instructions.***

Tables 2, 3, S10, S11 - please specify in the table legend which factors are adjusted for in ‘Adjusted 1’ results. When a p value is given, please specify the statistical test used to determine it in the table legend.

**Response:**

***Thank you; we have Table 2,3,S10 and S11 changed to “Analysed with multilevel mixed model adjusted for centre as random factor and period …”***

References:

Please ensure that journal name abbreviations match those found in the National Center for Biotechnology Information (NCBI) databases (<https://eur02.safelinks.protection.outlook.com/?url=http%3A%2F%2Fwww.ncbi.nlm.nih.gov%2Fnlmcatalog%2Fjournals&data=05%7C02%7Chelena.backman%40regionorebrolan.se%7Cd0543685f40f42a8dde008dc736bf7ac%7Caece5b1982274c2782181aea120ec062%7C0%7C0%7C638512154825647266%7CUnknown%7CTWFpbGZsb3d8eyJWIjoiMC4wLjAwMDAiLCJQIjoiV2luMzIiLCJBTiI6Ik1haWwiLCJXVCI6Mn0%3D%7C0%7C%7C%7C&sdata=2%2BndRsDFmevMPmrXjRtEVLyUoLJyoWoCijYDkWccNdk%3D&reserved=0>), and are appropriately formatted and capitalised.

Please also see <https://eur02.safelinks.protection.outlook.com/?url=https%3A%2F%2Fjournals.plos.org%2Fplosmedicine%2Fs%2Fsubmission-guidelines%23loc-references&data=05%7C02%7Chelena.backman%40regionorebrolan.se%7Cd0543685f40f42a8dde008dc736bf7ac%7Caece5b1982274c2782181aea120ec062%7C0%7C0%7C638512154825647266%7CUnknown%7CTWFpbGZsb3d8eyJWIjoiMC4wLjAwMDAiLCJQIjoiV2luMzIiLCJBTiI6Ik1haWwiLCJXVCI6Mn0%3D%7C0%7C%7C%7C&sdata=fxT0fXyO3hygxcabi2xyjtdEgFmHmzMOrKJCz0QLARc%3D&reserved=0> for further details on reference formatting.

Where website addresses are cited, please specify the date of access.

**Response:**

***Thank you; The PLoS style format provided for Endnote has been used: (***[***https://endnote.com/downloads/styles/plos-public-library-of-science-all-journals/***](https://endnote.com/downloads/styles/plos-public-library-of-science-all-journals/)***).***

***The abbreviations have been checked and changed for several references. For the website the access date is stated as ”cited..xx” according to the style formatting above but can be changed to “access date..XX” upon request.***

***Please let us know if we should download an alternative style.***

Comments from Reviewers:

Reviewer #1: I would like to thank the authors for their efforts to address my questions. I have no additional comments to make.

**Response:**

***Thank you, we appreciate the constructive comments.***

Reviewer #2: Thank you to the authors for addressing my previous comments well. The only minor issue I had was on my initial review's point 1. Personally I would recommend adding 'a number of' before 'clusters' but this can be improved at the post-acceptance proofing stage. I had no other issues to raise.

**Response:**

***Thank you; we have added in abstract ”a number of..” according the reviewers comment.***

Reviewer #3: Thank you for responding to each of our comments with such care. I have no further comments to add.

**Response:**

***Thank you for valuable constructive comments.***

Any attachments provided with reviews can be seen via the following link:

__________________________________________________

In compliance with data protection regulations, you may request that we remove your personal registration details at any time. (Use the following URL: <https://eur02.safelinks.protection.outlook.com/?url=https%3A%2F%2Fwww.editorialmanager.com%2Fpmedicine%2Flogin.asp%3Fa%3Dr&data=05%7C02%7Chelena.backman%40regionorebrolan.se%7Cd0543685f40f42a8dde008dc736bf7ac%7Caece5b1982274c2782181aea120ec062%7C0%7C0%7C638512154825803514%7CUnknown%7CTWFpbGZsb3d8eyJWIjoiMC4wLjAwMDAiLCJQIjoiV2luMzIiLCJBTiI6Ik1haWwiLCJXVCI6Mn0%3D%7C0%7C%7C%7C&sdata=4lF56tFyiQnp8REA3OF1wu08SzxK1wIdQsF0QB6heTw%3D&reserved=0>).

Please contact the publication office if you have any questions.
